# Ultrafast Response and Threshold Adjustable Intelligent Thermoelectric Systems for Next-Generation Self-Powered Remote IoT Fire Warning

**DOI:** 10.1007/s40820-024-01453-x

**Published:** 2024-07-10

**Authors:** Zhaofu Ding, Gang Li, Yejun Wang, Chunyu Du, Zhenqiang Ye, Lirong Liang, Long-Cheng Tang, Guangming Chen

**Affiliations:** 1 College of Materials Science and Engineering & College of Civil and Transportation Engineering, Shenzhen University, Shenzhen, 518055 People’s Republic of China; 2 College of Material, Chemistry and Chemical Engineering, Key Laboratory of Organosilicon Chemistry and Material Technology of MoE, Hangzhou Normal University, Hangzhou, 311121 People’s Republic of China

**Keywords:** Thermoelectric, Self-powered, IoT fire warning, Ultrafast response, Threshold adjustable

## Abstract

Fire warning is vital to human life, economy and ecology. However, the development of effective warning systems faces great challenges of fast response, adjustable threshold and remote detecting. Here, we propose an intelligent self-powered remote IoT fire warning system, by employing single-walled carbon nanotube/titanium carbide thermoelectric composite films. The flexible films, prepared by a convenient solution mixing, display p-type characteristic with excellent high-temperature stability, flame retardancy and TE (power factor of 239.7 ± 15.8 μW m^−1^ K^−2^) performances. The comprehensive morphology and structural analyses shed light on the underlying mechanisms. And the assembled TE devices (TEDs) exhibit fast fire warning with adjustable warning threshold voltages (1–10 mV). Excitingly, an ultrafast fire warning response time of ~ 0.1 s at 1 mV threshold voltage is achieved, rivaling many state-of-the-art systems. Furthermore, TE fire warning systems reveal outstanding stability after 50 repeated cycles and desired durability even undergoing 180 days of air exposure. Finally, a TED-based wireless intelligent fire warning system has been developed by coupling an amplifier, analog-to-digital converter and Bluetooth module. By combining TE characteristics, high-temperature stability and flame retardancy with wireless IoT signal transmission, TE-based hybrid system developed here is promising for next-generation self-powered remote IoT fire warning applications.

**Supplementary Information:**

The online version contains supplementary material available at 10.1007/s40820-024-01453-x.

## Highlights

- The flexible single-walled carbon nanotube/titanium carbide composite films exhibit excellent thermoelectric (TE), high-temperature stable and flame-retardant properties.
- The assembled TE device achieves an ultrafast fire warning response time of ~ 0.1 s with a threshold voltage of 1 mV.
- The fire warning device demonstrates exceptional repeatability and long-term stability.
- The designed intelligent system is promising for next-generation self-powered remote IoT fire warning applications.

**Supplementary Information:**

The online version contains supplementary material available at 10.1007/s40820-024-01453-x.

## Graphical abstract

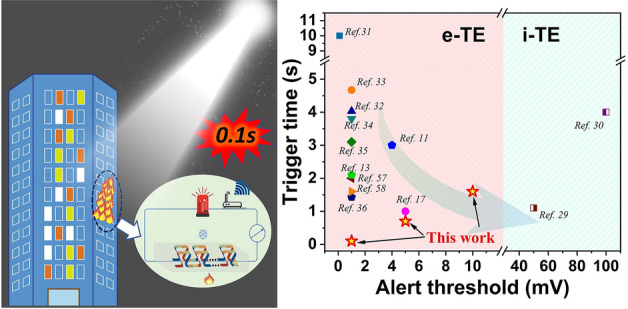

## Introduction

Fire disasters have killed thousands of lives and led to tremendous economic loss each year, and have always become a serious problem for long time. In recent decades, fire alarming has garnered special attention, particularly with the widespread application of electrical appliances and the mass use of flammable materials in buildings and decorations [1]. The rise of the Internet of Things (IoT) era has further heightened the necessity for intelligent fire warning materials to proactively address fire hazards in the infancy [2, 3]. In this regard, the exploration of temperature/heat detection to realize early fire warning has emerged to offer a promising solution [4, 5]. Consequently, various techniques based on temperature-sensitive materials, including thermistor [6], thermoelectric (TE) [7], thermo-chromic [8] and thermo-responsive phase-change/shape-change principles [9, 10], have been developed. Very recently, TE materials have aroused special interest due to their unique self-powered ability without the requirement of external power sources, wide application environments of both indoor and outdoor open conditions, fast response time and high repeatability/durability [11–17].

When being subjected to a temperature difference caused by flame, TE-based fire warning materials generate thermo-voltage signals benefiting from their inherent Seebeck effect, which enables timely triggering of fire alarm signals [2, 7]. To achieve a large output voltage, p- and n-type TE materials, which form p–n couples, are usually alternately assembled in series into TE devices (TED) [12, 18–20]. The theoretical output voltage is determined by the equation (*U* = *N*_p_ *S*_p_ Δ*T* + *N*_n_ |*S*_n_| Δ*T*), where *N* is the number of p- or n-type TE unit, *S* denotes the Seebeck coefficient of p-type or n-type TE materials and Δ*T* is the temperature difference between the hot and cold parts [21–28]. Hence, the performance of the TE materials plays a crucial role in determining the responsiveness of fire warning systems [7]. Additionally, during fire warning process, a threshold voltage is typically established based on the intrinsic TE capability of the material. When the voltage, generated by the detected flame temperature, reaches the predetermined threshold voltage, the fire warning signal will be triggered [2]. As a consequence, the trigger or response time of the fire warning system mainly depends on the TE properties and the designated threshold voltage. Besides high TE performance, flame retardancy, high-temperature stability and long durability in air are also required for the material design in fire warning applications.

At present, the study of TE-based fire alarming is at the early stage, and only few tentative reports have been found in a literature survey [29–36]. For example, Ashcheulov et al.[37] reported an anisotropic TE sensor, composed of eutectic CdSb–CoSb alloy, to map the areas in danger of fire in coal mines. Peng and co-workers fabricated a flexible film of MXene and aramid nanofiber, which triggered the fire warning system within 10 s when exposed to fire [31]. In these preliminary investigations, the TE and corresponding fire alarming properties are low. Additionally, the flame retardancy performance, the high-temperature stability and the long-term durability have been seldom taken into account. Theoretically, through judicious design, such performances can be merged into a single composite system. Single-walled carbon nanotubes (SWCNTs) have witnessed significant advances serving as TE constituents due to their unique 1D nanostructure, good electrical conductivity and medium Seebeck coefficient [38–41]. However, their low flame retardancy hinders the use for fire warning. MXene, composed of 2D nanoplatelets with transition metal carbides, nitrides or carbonitrides, possesses super electrical conductivity (> 8000 S cm^−1^) [42], excellent flame retardancy as well as high thermal and air stabilities [43, 44]. Consequently, it is reasonable to deduce that simultaneous enhancements of both TE and flame retardancy can be achieved by employment of MXene into SWCNTs with homogeneous dispersion and strong interfacial interaction.

In this work, we have developed an intelligent self-powered remote IoT fire warning system, based on high-performance SWCNT/MXene TE composites. The p-type composites were prepared by convenient solution mixing, and the n-type ones were obtained by doping the p type using amine-rich polymer of polyethyleneimine (PEI). Flexible TEDs with cross-plane design were fabricated via assembling the p- and n-doped composite films in series. Importantly, the fire warning threshold voltages are tunable, and remote fast warning response can be realized. When the threshold voltage was set at 1 mV, the warning response time reached ~ 0.1, 0.6 and 1.9 s at flame distances of 0, 10 and 20 cm, respectively. Besides these exciting results, the fire warning repeatability and long-term durability of TED were also investigated. Finally, by combination with ADC (analog-to-digital converter), buzzer and Bluetooth module, an intelligent self-powered remote IoT fire warning system was developed. This study opens a new avenue to next-generation remote IoT fire warning scenarios based on TE principle with ultrafast response time, wide adjustable threshold voltage and alarming performances.

## Experimental Section

### Materials

SWCNT (diameter: 1–2 nm, length: 5–30 μm, purity: > 95.0 wt%) was purchased from XFNANO Materials Tech Co., Ltd. MXene (Ti_3_C_2_T_x_) fewer (single) layers of dispersion were purchased from Jinlin 11 Technology Co., Ltd. Polyethyleneimine (PEI) (molecular weight: 600, purity > 99%) and anhydrous ethanol were purchased from Shanghai Aladdin Bio-Chem Technology Co. Ltd., China. The pristine flammable sponge was purchased from Ningbo Shijie Cleaning Tools Co., Ltd. The filtration membrane is MXene dedicated membrane (Celgard 3501). All reagents were used as received unless otherwise specified. Deionized water was used in all the cases of the experiments.

### Preparation of p- and n-Type Freestanding Film and Assembling to the Device

**Fig. 1:**
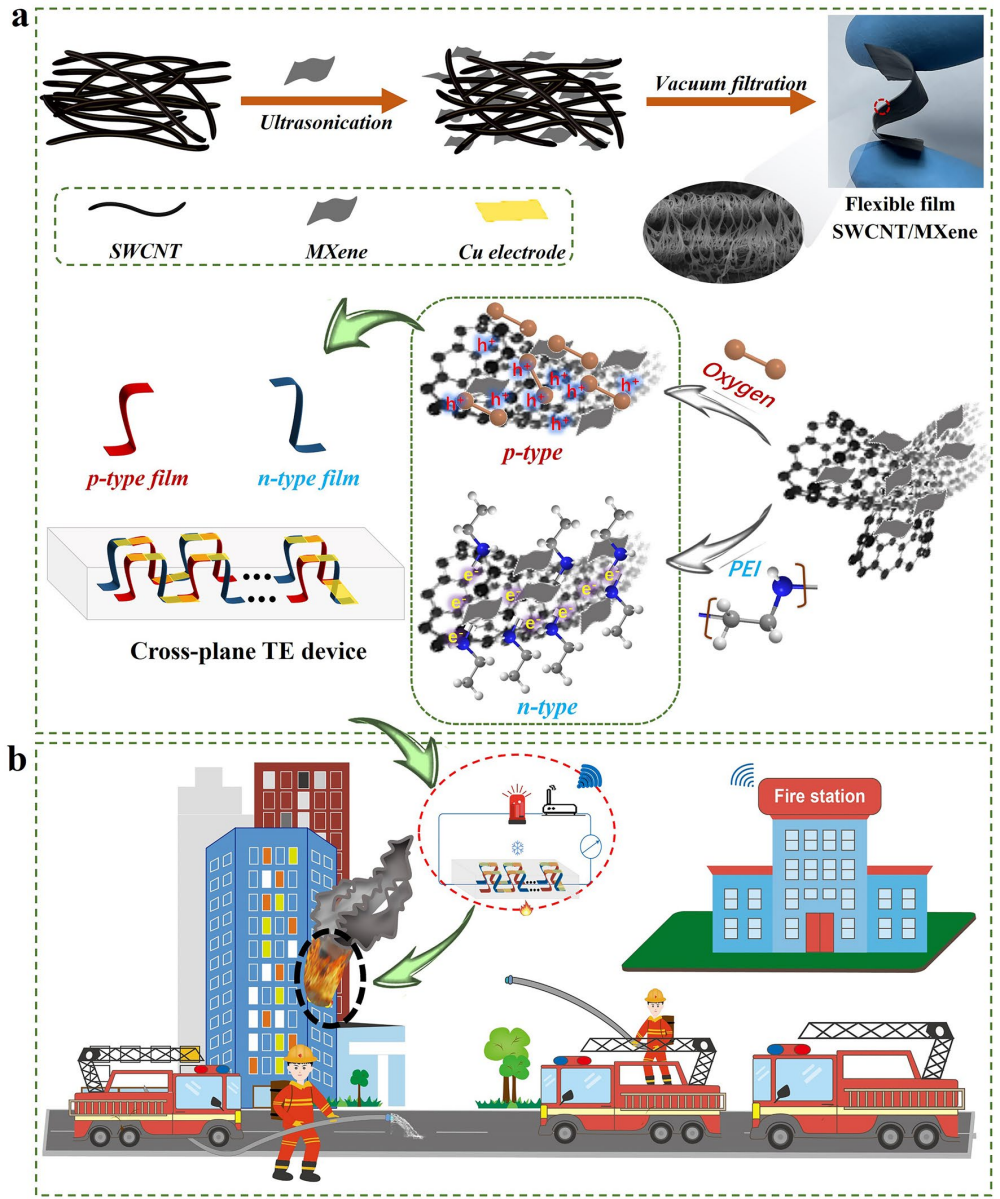
Material preparation, device assembly and application of SWCNT/MXene film-based TE IoT fire warning system. **a** Illustration of the preparation process and the brief doping mechanisms of p-type and n-type SWCNT/MXene composite film, and the assembled cross-plane TE device. **b** Conceptual diagram of next-generation intelligent remote IoT fire warning system

Carbon nanotubes and MXene were ultrasonically dispersed in ethanol, followed by vacuum filtration to obtain series of freestanding films with different mass ratio of SWCNT to MXene (from 10:1 to 10:7). The n-type film was obtained by soaking the p-type film in 1 wt% PEI aqueous solution for 12 h and then vacuum drying. In order to better obtain the temperature difference, a vertical structure design is adopted, with p–n sequentially connected, as shown in the device structure in Fig. 1. The middle is filled with insulation sponge, maintaining a good temperature difference between the cold and hot ends.

### Characterizations

The micromorphological observations and energy-dispersive X-ray spectroscopy (EDX) of the composite films were observed by a field emission scanning electron microscope (FESEM, Thermo APREO S) with an acceleration voltage of 5 kV. The X-ray diffraction patterns were tested by an X-ray diffractometer (XRD, Bruker D8 Advance, Germany) with Cu K*α* radiation (*λ* = 1.5406 Å) from 3° to 60° at a scanning rate of 5° min^−1^. The surface chemical composition of the films was analyzed via an X-ray photoelectron spectroscopy (XPS, ESCALAB 250XI, Thermo Fisher, USA). The Raman spectra of all the films were collected with a Raman spectrometer (Xplora Plus, HORIBA Scientific) using a laser with an excitation wavelength of 532 nm. The thermal decomposition behavior of the composite films was performed on a thermal analysis system (TA TGA-Q50) with a heating rate of 10 K min^−1^ and a temperature range of room temperature to 800 °C (in air atmosphere). A Fluke TiS55 + /TiS75 + thermal imager was used to monitor the temperature of the alcohol lamp in vertical burning tests.

### Mechanical Property and TE Performance Tests

The typical stress–strain curves were obtained using a tensile machine (SANS EUT4103) with a 50 N load at a rate of 5 mm min^−1^ in air under room temperature. The freestanding films were set to the dimensions of 18 μm (thickness), 30 mm (length) and 10 mm (width), with the gauge length set to 15 mm. All the samples were tested for five times. Thermoelectric properties were tested using an MRS-3 M thin film thermoelectric parameter test system with a vacuum environment (Wuhan Joule Yacht Science & Technology Co., Ltd, China). Three specimens were tested for each set of samples, and standard deviation was then calculated. TE output voltage was collected by using a homemade apparatus.

### Flame-Retardant Performance

The composite dispersion is obtained as above; then, the commercially available combustible polymer sponge is immersed in SWCNT/MXene 10:3 composite dispersion with a size of 9 × 2 × 0.5 cm^−3^. (The adsorption capacity of each sponge unit is 2 mg cm^−3^.) The flame-retardant sponges were obtained after drying in 60 °C for 4 h. In a typical combustion testing, alcohol lamps were used and all the samples have the same distance to flames.

### Fire Warning Performance

The TE devices were connected with an alarm device (HB414, Dongguan Daxian Instrument Equipment Co., Ltd., China) and burnt using an alcohol lamp, and the threshold was set in a broad range in 1–10 mV. The corresponding fire warning process was recorded using a camera.

### Thermal Conductivity Test

The thermal conductivity of the freestanding films was calculated by the formula: $k = \alpha \rho c_{p}$, where *α* is thermal diffusivity, *ρ* is density and *c*_*p*_ is heat capacity. *α* was measured using the transient electrothermal (TET) technique, where the sample was cut into narrow strips of about 350 μm width and 1 mm length, fixed by silver paste to minimize the thermal contact resistance (Fig. S1). The *C*_*p*_ was obtained by differential scanning calorimetry (DSC, NETZSCH404F3) analysis, and the density *ρ* was calculated using the formula *m* = *ρV*.

## Results and Discussion

### Fabrication of SWCNT/MXene TE Composites, TEDs for Next-Generation Remote IoT Fire Warning Scenario

The flexible SWCNT/MXene TE composite films were prepared via a convenient solution mixing of the homogeneous dispersions of SWCNTs and MXene with subsequent vacuum filtration, as shown in Fig. 1a. The prepared SWCNT/MXene composite films exhibited inherent p-type characteristics, resulting from the adsorbed oxygen located on the surfaces of SWCNTs and MXene, *i.e.*, hole-doping effect. The corresponding n-type films were successfully achieved by soaking the p-type SWCNT/MXene composite films in PEI (amine-rich dopant) solution overnight [11]. The electron-donating capability of the amine groups in PEI leads to effective n-type doping, displaces the surficial oxygen molecules and enables electron transfer to SWCNTs and MXene [45]. Figure S2 demonstrates the remarkable flexibility of the SWCNT/MXene composite film, since it can be facilely bent and wound around a cylindrical object with a radius as small as 1.5 mm. In addition, the mechanical properties were also evaluated by tensile testing. In Fig. S3, both the p-type and n-type composite films exhibit greatly enhanced elastic moduli and breaking strains compared to the pure SWCNT film. To efficiently utilize the thermal energy, a vertical-type TE device, where the temperature gradient is perpendicular to the device planes, was fabricated by serially connecting p- and n-type films with a temperature-resistant silicone adhesive as the supporting material [46]. By judiciously adjusting the number and size of p–n couples, various TE devices could be conveniently obtained [19].

Figure 1b illustrates the conceptual diagram of next-generation intelligent remote IoT fire warning system. A flagship system not only overcomes the traditional shortage of flammability feature, but also complies with the next-generation criteria, *i.e.*, (i) triggering rapid response, (ii) keeping structure stability, (iii) working efficiently under complicated environments and (iv) manipulating in large-scale production [2]. Besides, remote wireless signal transmission is also a key factor in the design strategy for next-generation fire warning system, especially in the era of IoT.

### Morphological and Microstructural Characterizations

The morphological characteristics of the pure SWCNTs, MXene and their composites are characterized by scanning electron microscope (SEM). In Fig. S4, the pure SWCNT and MXene display typical 1D and 2D characteristics, respectively. In the composites, the MXene nanoplatelets are closely attached on the surfaces of the 1D SWCNT frameworks, facilitated by interfacial interactions. Moreover, a cross-sectional SEM image demonstrates a distinct interconnected layered ordered structure for the pure SWCNT film, mainly originating from the strong *van der Waals* attractions of adjacent nanotubes induced during the vacuum filtration procedure [47]. The pure MXene film exhibits a dense and compact cross section. Notably, in the composite film, the SWCNT network-supported layered architecture is well maintained, with the MXene flakes evenly distribute in the networks. The distribution of MXene nanoplatelets within the SWCNT network was also examined by the relative element content through corresponding EDS analyses, as shown in Fig. S5 and Table S1. EDS spectra reveal the uniform distribution of Ti, F, C and O elements in the composite film, indicating the homogeneous dispersion of MXene flakes and SWCNTs.

Raman spectra, XRD patterns and XPS spectra were collected to analyze the elemental/structural information. As shown in Fig. S6, typical Raman spectrum of SWCNT involved D band and G band. The band at 1332 cm^−1^ results from the lattice defect of C atoms caused by the destruction of hexagonal *sp*^2^ hybridization, while the characteristic band at 1584 cm^−1^ represents the in-plane stretching vibration. Note that the value of the intensity ratio of these two bands, *I*_D_/*I*_G_, is usually adopted to quantitatively evaluate the defect content. In the composite, the *I*_D_/*I*_G_ is low, indicating with little defects exist. Meanwhile, the increasing ratio relative to the neat SWCNTs suggests that the presence of the interfacial interaction between SWCNTs and MXene platelets [48]. Indeed, the blueshift of the G band also implies the effective electron transfer, demonstrating to the existence of SWCNT/MXene interfacial interactions [49].

To further elucidate the interactions between MXene and SWCNTs, X-ray diffraction (XRD) analysis was performed, and the interlayer spacing was calculated using Bragg’s law (*λ* = 2*d* sin*θ*). In Fig. S7, the diffraction peak at approximately 3.90° corresponds to a pure SWCNT basing spacing of 2.26 nm. Upon incorporation of MXene, the characteristic peak gradually shifts to higher angles with increasing SWCNT: MXene mass ratio from 10:1 to 10:7, which corresponds to the basal spacings of 2.26, 1.34, 1.29, 1.27 and 1.23 nm, respectively. This reduction in basing spacings indicates more compact stacking and stronger interactions, thus benefiting the TE performance [48]. Figure S8a presents the XPS survey spectra of the samples. Evidently, the presence of MXene results in an enhancement in the binding energy of characteristic peaks corresponding to Ti, C, O and F elements. The XPS C 1*s* spectra of the pristine SWCNT film exhibit distinct characteristic peaks at 284.8, 285.7 and 287.5 eV, which are attributed to C = C/C–C, C–O and C=O bonds, respectively (Fig. S8b) [48]. In the composites, these three binding energies experience a downward shift to 284.6, 285.5 and 286.3 eV, respectively, providing an evidence for the presence of interfacial interactions and partial charge carrier transfer between SWCNT and MXene (Fig. S8c) [32].

### High-Temperature Stability and Flame-Retardant Behaviors

**Fig. 2:**
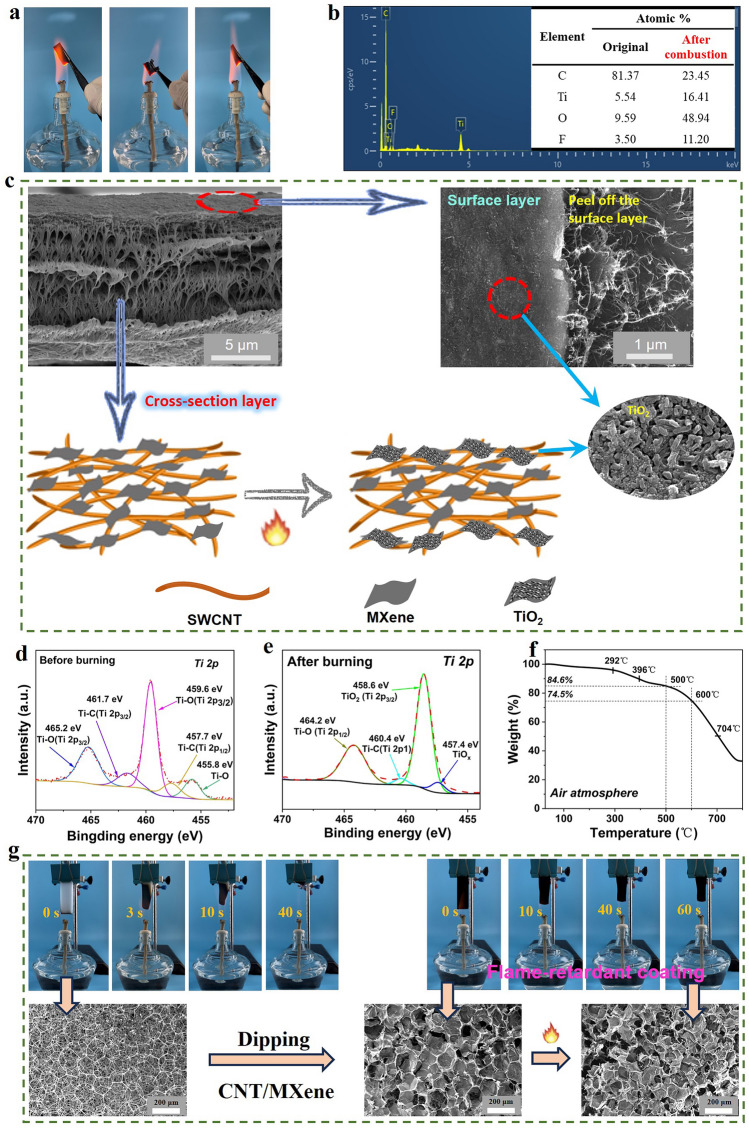
Flame retardancy of SWCNT/MXene composites SWCNT, MXene and (ii) SWCNT/MXene composite film. **a** Typical burning process of the pure, showing excellent flame resistance and good structural integrity. **b** EDS spectra of the burned composite. The inset image shows the relevant element content of the composite before and after combustion.** c** SEM images and schematic diagram of the SWCNT/MXene composite before and after combustion. **d, e** XPS Ti 2*p* spectra of the composites before and after burning. **f** TGA curve of the composite in air atmosphere. **g** Typical burning process of the pristine uncoated sponge and the SWCNT/MXene-coated sponge. The corresponding SEM images for the pristine sponge, the SWCNT/MXene-coated sponge before and after combustion

Considering practical application scenario, the flame retardancy is a crucial index for TE-based fire warning materials. The burning tests were conducted to visually evaluate the flame-retardant performance of the as-prepared SWCNT/MXene films. As depicted in Fig. 2a and Video S1, the composite film maintained its structural integrity even after being exposed to a fire for 60 s, with a flame temperature exceeding 500 °C (Fig. S9). SEM images reveal the microstructural evolution before and after combustion. Distinctly, the pure SWCNTs experienced partial surface carbonization, resulting in a smaller diameter compared to the pristine SWCNTs Fig. S10a). Interestingly, upon tearing off the surface with adhesive tape, the internal morphology of SWCNTs are similar to that of the unburned SWCNT film (Fig. S10b), indicating its superior high-temperature stability [5, 50]. For pure MXene paper, no ignition was observed after 60-s combustion, due to the inorganic framework. Only a color transition from black to gray occurred, resulting from the thermal oxidation at high temperature. The EDS spectra (Fig. 2b) compares the relative content of elements before and after combustion of the composite film. The relative content of oxygen (O) element increases significantly, whereas the relative content of titanium (Ti) element reduces, demonstrating the abundant oxidation of MXene after combustion [51]. From the perspective of surface morphology, the internal morphology of the burned composite film was well remained, being consistent with that being peeled off from the burned film’s surface using adhesive tape (Fig. 2c). The composite excellent flame retardancy can be mainly attributed to the formation of TiO_2_ flame-retardant layer through partial thermal oxidation of MXene and the exceptional thermal stability of SWCNT [52]. This TiO_2_ layer served as an effective protective barrier, preventing further oxidation of the internal MXene (Fig. S10c, d). Additionally, the presence of TiO_2_ located in the burned composite film is also verified by XPS spectra, as shown in Fig. 2d, e [34].

To further assess the high-temperature stability performance, a thermo-gravimetric (TGA) analysis was carried out. As depicted in Fig. S11, the TGA curve of the pure SWCNTs shows a one-step degradation process, with the maximum weight loss rate occurring at 680 °C, suggesting its good thermal stability. In contrast, the pure MXene exhibits a complex decomposition process with three stages in the TGA curve. In the initial stage below 200 °C, water vapor evaporation occurs along with the detachment of F/O elements, resulting in a decrease in weight. The second stage, ranging from 200 to 370 °C, involves the oxidation process of Ti in MXene, leading to the conversion into TiO_2_ and an increase in weight. The third stage corresponds the destruction of the Ti_3_C_2_ layer, where the impact of degradation outweighs the oxidation, resulting in a weight decrease. Comparatively, the thermal decomposition of the composite film is more complex and can be divided into three stages as well. Two maximum decomposition temperatures occur at 396 and 704 °C, respectively (Figs. 2f and S10b). Compared to the maximum weight loss temperature of the pure SWCNT at 680 °C, the composite material reveals a higher maximum weight loss temperature of 704 °C, indicating a superior high-temperature stability. This enhancement can be attributed to the synergistic interactions between the SWCNTs and MXene. Furthermore, the resultant composite film exhibits a mass loss of only 25.5% at 600 °C, when most materials underwent combustion. This remarkable improved thermal stability is of great significance for its potential utilization under high-temperature harsh environments.

To visualize more clearly of the excellent flame retardancy, the SWCNT/MXene composite was utilized as a flame-retardant coating for validation purpose. In a typical vertical burning process (Fig. 2g and Video S1), a commercial melamine sponge was employed as comparison. The uncoated sponge was completely destroyed under the flame and burned completely within 40 s, leaving no residues. In sharp contrast, the sponge coated with SWCNT/MXene composite layer, where the immersion coating was verified by SEM images (Fig. 2g), demonstrates the excellent ability to resist ignition during a 60-s burning process, with only minimal volume shrinkage. Furthermore, no obvious damage can be recognized for the porous structure of the sponge coated with SWCNT/MXene after combustion, confirming the excellent flame-retardant property of the SWCNT/MXene. Meanwhile, the composite sponge reveals a resilience even under the exposure to flames, with little structural damage observed after combustion (Fig. 2g).

In addition, the limiting oxygen index (LOI), being an important parameter for assessing flame retardancy, was employed to evaluate the flame-retardant performance of the SWCNT/MXene-coated sponge. The LOI value for the uncoated sponge is 32.9%. After coating with SWCNT/MXene, it increases distinctly to 36.0%, also demonstrating enhanced flame retardancy [50].

### TE Performances of the Composite Films and TE Devices

**Fig. 3:**
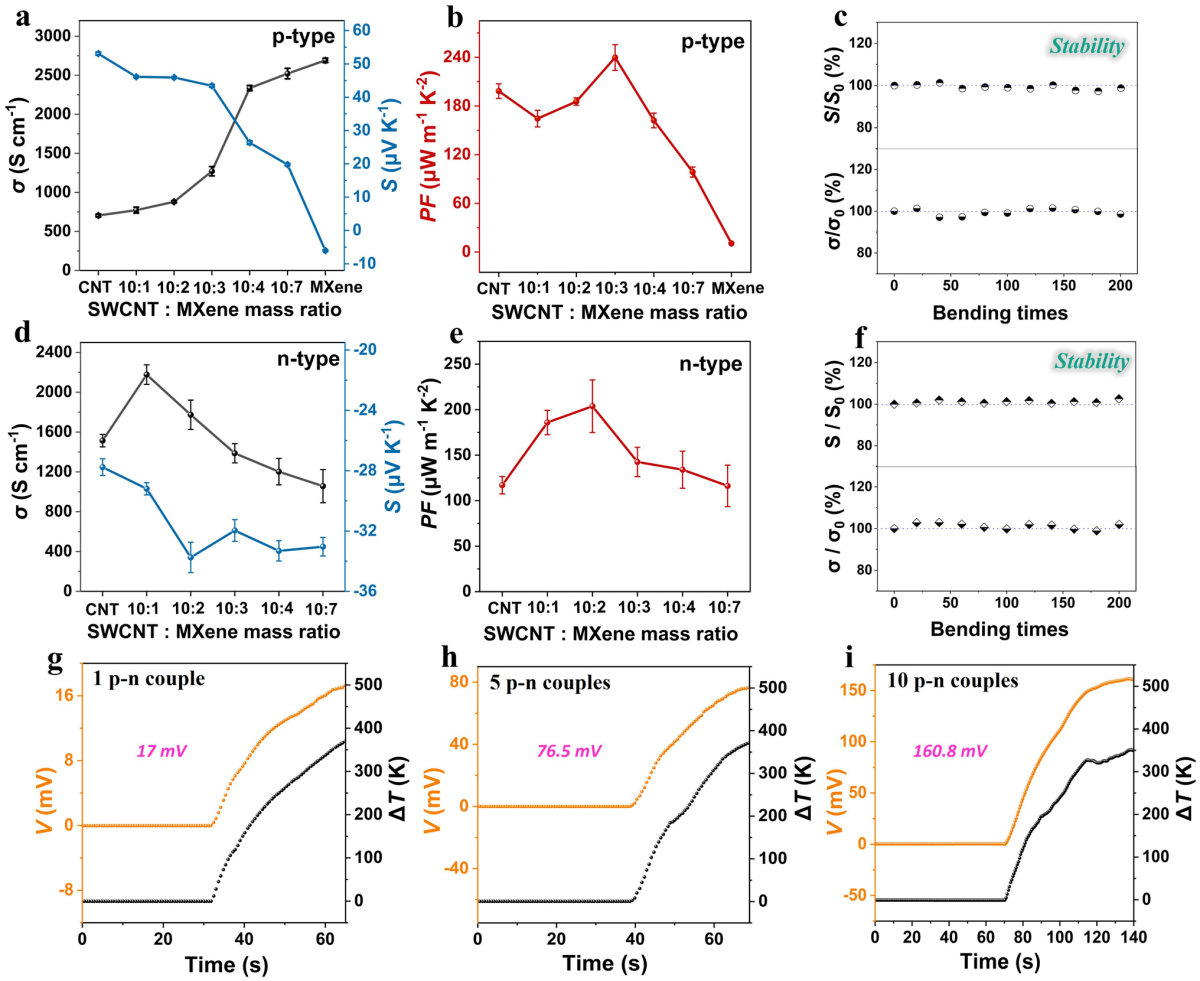
TE properties of composite films and their devices. **a** Electrical conductivity, Seebeck coefficient and **b** the corresponding power factor with various ratio of p-type SWCNT/MXene films. **c** Stability of TE properties of p-type films with the mass ratio of 10:3 after multiple bending cycles. **d** Electrical conductivity, Seebeck coefficient and **e** the corresponding power factor with various ratio of n-type SWCNT/MXene films. **f** Stability of TE properties of n-type films with the mass ratio of 10:2 after multiple bending cycles. Output voltages using alcohol lamp as heating source for the TED with **g** 1, **h** 5 and **i** 10 p–n couples

The TE properties of the prepared p- and n-type SWCNT/MXene composite films were evaluated. To explore the optimal mass ratio of SWCNTs to MXene, the corresponding TE properties, including *σ*, *S* and the calculated *PF* of the resultant p-type SWCNT/MXene composite films, are plotted in Fig. 3. In Fig. 3a, the *S* of the pristine MXene was negative, indicating its dominant internal charge carriers are electrons. By combining the intrinsic high *σ* of MXene with the intrinsic large *S* of SWCNTs, a remarkable increase in *σ* and a notable decrease in *S* are observed with the increase of MXene content. As a result, an optimal *PF* of 239.7 ± 15.8 μW m^−1^ K^−2^ appears at SWCNT: MXene mass ratio of 10: 3 (Fig. 3b), being higher than that of the pristine SWCNT, MXene and the reported SWCNT/MXene-based systems (Table S2). The substantially enhanced *PF* is perhaps ascribed to three key factors: the inherent good TE properties of SWCNT and MXene, the efficient interfacial interactions between SWCNT and MXene (as evidenced by Raman spectra, Fig. S6), and the novel layered structure (Fig. S4). Subsequently, in order to obtain the matching n-type films, all prepared p-type SWCNT/MXene composite films were soaked in a 1% PEI aqueous solution for 12 h, and their corresponding TE properties are revealed in Fig. 3d, e. It can be seen that the *σ* initially increases and then sharply reduces, while the *S* enhances monotonically with increased MXene content. Thus, the PEI-doped composite film achieves an optimal *PF* value of 202.0 ± 28.9 μW m^−1^ K^−2^ at SWCNT: MXene mass ratio of 10:2, around 20 times of that of the pristine MXene. Besides the super flexibility of both p- and n-type composite films, the mechanical stability of TE performance under bending deformations is also evaluated. As depicted in Fig. 3c, f, both *σ* and *S* are well preserved after repeated bending cycles, indicating the desired mechanically stability for both p- and n-type films.

To further evaluate the heat transfer behavior of the films, we conducted thermal conductivity tests [53]. Firstly, the thermal diffusion coefficient *α* was obtained by TET technology, and the heat capacity *c*_*p*_ were obtained using DSC. The relevant parameters are shown in Table S3. The decrease in thermal conductivity is caused by the increased phonon scattering due to doping. In addition, the dopant material itself has a lower thermal conductivity than SWCNT. In the end, the calculated *ZT* value for the optimal component (SWCNT: MXene 10:3 wt%) is 9.27 × 10^–3^.

To take full advantage of high-performance composite films, cross-plane structured TEDs are designed where the heat flows vertically to the device planes [49, 54, 55]. The p–n couples with varying numbers were alternately connected in series, and a temperature-resistant silicone adhesive was applied as supporting material. Given the excellent high-temperature resistance of the p–n TE films, the assembled TEDs were applied to harvest thermal energy of the flame of an alcohol lamp. When the TEDs were positioned on the top of an alcohol lamp, real-time voltage variations were recorded, as illustrated in Fig. 3g-i. The voltages of all the three TEDs vary synchronously with Δ*T*, demonstrating their rapid and sensitive temperature responsiveness. Notably, the largest generated voltages for TED-1pn, TED-5pn and TED-10pn were measured to be 17, 76.5 and 160.8 mV, respectively. The deviations from the expected theoretical values are primarily attributed to the inevitable contact electrical and thermal resistances inside the TEDs.

### Fire Warning Performance

**Fig. 4:**
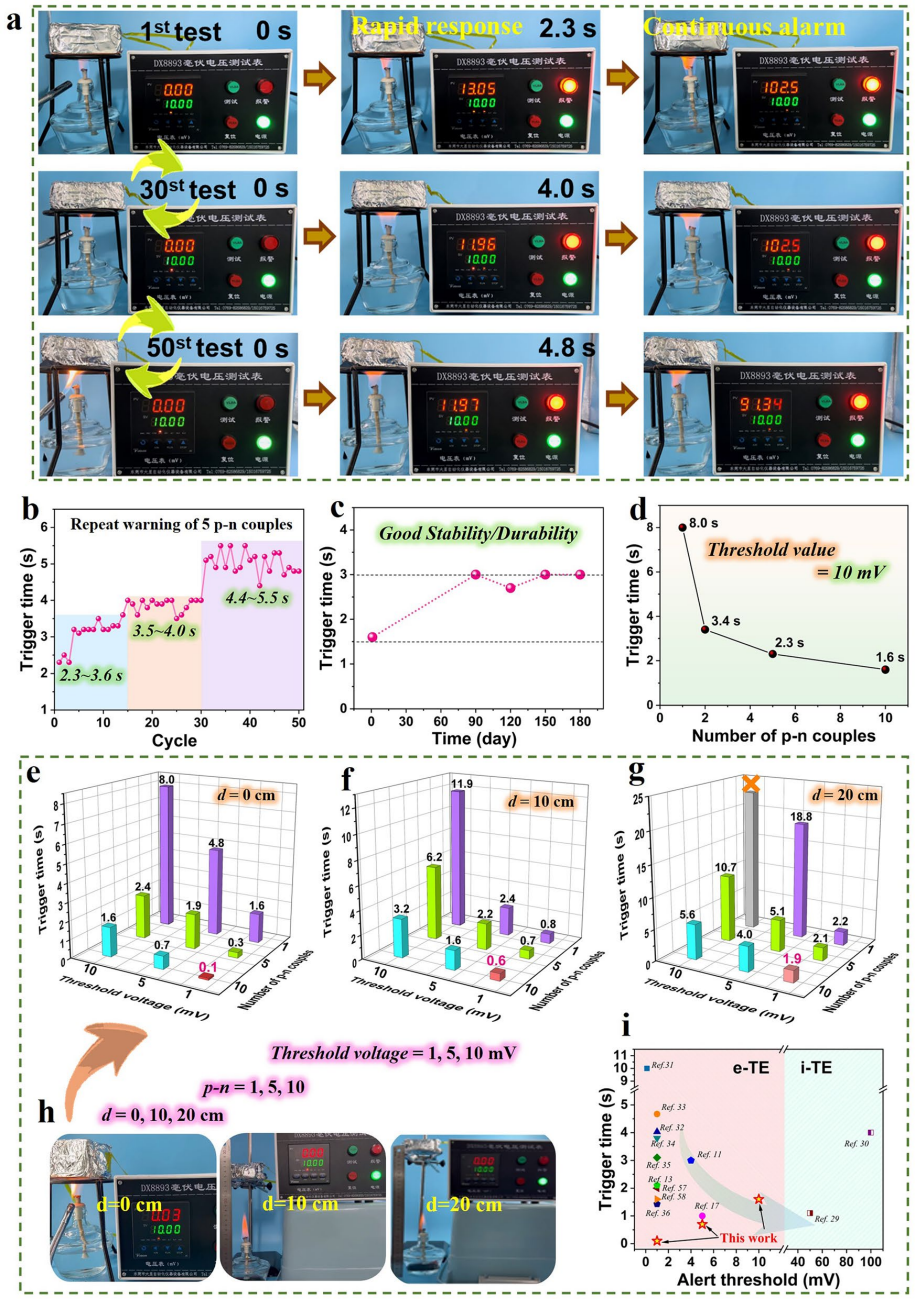
Fire warning performance of devices. **a** Repeated fire warning tests for 50 times with TED-5pn couples. **b** Effect of the number of cyclic uses on trigger time. **c** Environmental tolerance with TED-10pn couples. **d** Trigger time *vs* the number of p–n couples. **e–h** Impact of threshold voltage, number of p–n couples, and the distance between the device and the outer flame on the performance of fire warning. **i** The comparison of the trigger time of TEDs for fire warning applications

In order to explore the fire warning performance, the TED was directly connected to a millivolt voltage alarm. When the TED was exposed to fire, the temperature increase would result in a voltage due to the Seebeck coefficient. If the output voltage exceeds the set threshold voltage value, the millivolt voltage signal will be triggered. In Fig. 4a, the corresponding warning process is directly observed by the photos, where 10 mV is set as the early warning threshold voltage for TED-5pn. In the first cycle, the fire warning trigger time is only 2.3 s, which is much faster than that of many reported TE fire warning and other warning materials [56]. The fast and sensitive fire warning performance may be attributed to the admirable TE characteristics (large *S*, Fig. 3) of both p- and n-type SWCNT/MXene composite films. And the fire alarming signal can last for a long period. More importantly, being intermittently exposed to alcohol lamp flame, the TED fire warning reported here can be repeatedly used for many times (30 and 50 times shown in Fig. 4a) because of their good thermal stability, high-temperature stability and flame retardancy. It can be observed that the TED-5pn can quickly trigger the alarm device at the 30th and 50th cycles with short time of 4.0 and 4.8 s, respectively. Besides the video screenshots shown in Fig. 4a, Video S2 clearly presents the whole alarming process.

Considering the extreme importance of the repeated use and the effectiveness of fire alarming system for long-term durability, we studied the effects of the number of cyclic alarming, the service time and the number of the p–n couples on the trigger time (Fig. 4b-d). In Fig. 4b, the trigger time is 2.3–3.6 s within the first 15 cycles. Then, it gradually increases to 3.5–4.0 s during the 15–30 cyclic use, and eventually 4.4–5.5 s between 30th and 50th alarming. Hence, we conclude that even after 50 cycles, the trigger time is short and remains within 5.5 s, demonstrating its stable and repeatable fire warning capability. This is consistent with that drawn from Fig. 4a. Additionally, Fig. 4b shows that the trigger time displayed an essential increasing trend with relatively minor fluctuations within certain cycle intervals (4–15, 15–30 and 30–50 cycles). Figure 4c confirms the effectiveness and stability of the long-term service for the TE-based fire warning device. Obviously, during 180 days for the present study, the TED exhibits a good stability or durability. Indeed, although the trigger time extends to some extent, the absolute value is very short. As for the TED fabricated by 10 p–n couples (TED-10pn), the trigger time increases from 1.6 to 3 s, revealing the maintaining of the high sensitivity and effectiveness. Therefore, Fig. 4b and c strongly demonstrates the excellent durability, stability and effectiveness for the long-term service.

From the viewpoints of various application scenarios and diverse requirements, the trigger time is defined by the TED performance, the distance between the TED and the outer flame, and the set threshold voltage. For a given p–n couples and device structure, the TED performance depends on the number of p–n couples. Figures 4d and S12 display that the trigger time or response time for the present TEDs becomes progressively shorter, as the number of p–n couples increases from 1 to 10. Notably, when the threshold voltage is set as 10 mV, TED-10pn shows a remarkably short trigger time of only 1.6 s, surpassing that of the majority of available publications to date. Figure 4e-h presents the overall dependence of the trigger time with these three parameters, *i.e.*, the distance between TED and outer flame, threshold voltage and number of p–n couples. First, all these factors greatly affect the fire warning performance. A long distance between TED and outer flame means a low temperature for the TED hot side, a low-temperature gradient established, and hence, the generated voltage is low. At a set threshold, a long time is required to reach a certain for a long distance. As revealed in Figs. 4e–h, S13, and Video S3, when the threshold is constant, the trigger time obviously increases with a longer distance (from 0 to 20 cm). A high threshold voltage directly relates to a long period to reach the set voltage and thus corresponds to a long trigger time. As comparison, a low threshold voltage enables faster fire warning. At present, for the majority of reported materials, the threshold voltage is typically set as 1 mV [57, 58] (Fig. 4i), possibly due to the compromise of the TE performance and high-temperature resistance required for fire alarm materials. Meanwhile, with the increase of the number of p–n couples, the trigger time is significantly shortened due to the enhanced TE efficiency. As for the present work, when the threshold voltage is 1 mV for TED-10pn, the corresponding trigger time gradually reaches as short as ~ 0.1, 0.6 and 1.9 s at the distances of 0, 10 and 20 cm, respectively. Compared to most research, such as the gas/smoke sensors with a trigger time of ~ 100 s [59], this TED-based early-stage warning system provides much more timely response to fire hazards.

Wide range of threshold or alert voltage and fast alarming are vital in practical fire alarming scenarios. Indeed, high alert threshold voltages, such as 10 mV, have seldom been employed for e-TE materials (the dominant carriers are electrons or holes) due to their limited TE performance, while high voltage can be chosen for i-TE with large Seebeck coefficients (ions serve as the main carriers). Figure 4i and Table S4 further compare the fire alarming performance between our work and various reported literatures. On the one hand, the TED systems assembled by SWCNT/MXene composite developed here show outstanding warning performance at each of the alert threshold voltages of 1, 5 and 10 mV. All of the trigger time are low. In particular, in sharp contrast to the trigger time of higher than 1 s in the previous publications, our work verifies an ultrafast fire warning response of only ~ 0.1 s at the alert threshold voltage of 1 mV. On the other hand, the alert voltage is tunable and can be set as high as 10 mV, which is among the largest for e-TE materials. As a whole, the ultrafast trigger time and the wide adjustable alert threshold voltages enable our TEDs based on SWCNT/MXene composites very promising for the next-generation remote IoT fire alarming applications.

### Self-Powered Intelligent Remote IoT Warning System for Building Application Scenario

**Fig. 5:**
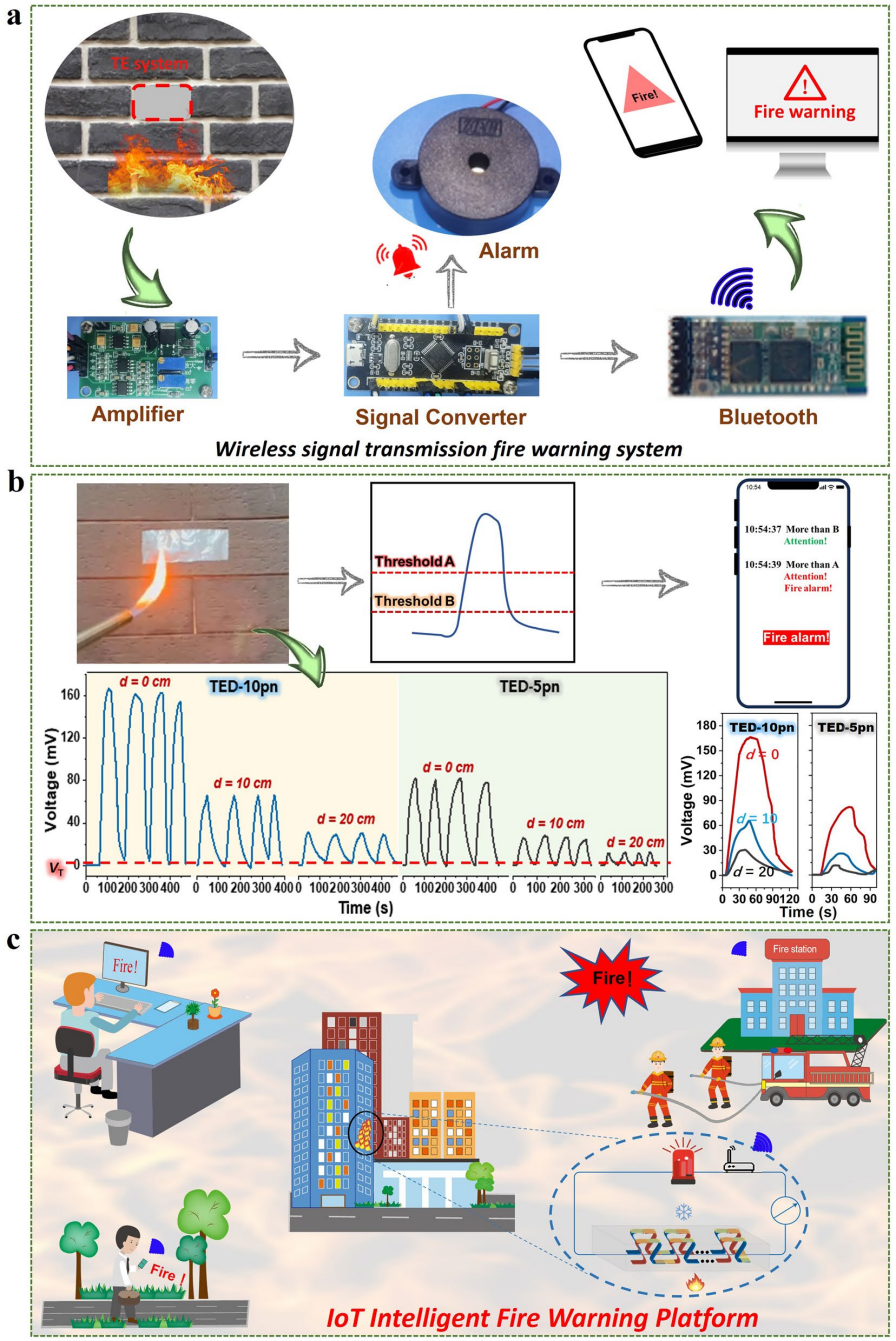
TE-based self-powered intelligent remote IoT fire warning system. **a** Schematic diagram of the designed self-powered intelligent wireless signal transmission for fire warning system.** b** Real-time voltage curves of TE fire warning system embedded in the building wall at different flame distances. **c** Proof of concept of an IoT intelligent fire warning platform for urban buildings based on the high-performance SWCNT/MXene TE composites

In order to take full advantage of the excellent fire warning properties of the assembled TEDs, we propose a self-powered intelligent IoT wireless TE fire warning system to alert firefighters and individuals. As illustrated in Fig. 5a, this system comprises a self-powered thermal-to-electricity conversion module (TED) for generating thermo-voltage when exposed to high temperatures, an amplifier for processing voltage signals, an ADC (analog-to-digital converter) for signal conversion, a buzzer and a Bluetooth module for remote signal transmission (Fig. S14). When the output thermo-voltage signal exceeds the warning trigger threshold voltage, the alarm TED is rapidly activated for alerting. And simultaneously, the fire alarm signal is remotely transmitted to firefighters and individuals via a wireless Bluetooth module. As illustrated in Fig. 5b, we embedded the system into building wall to conduct simulating flame tests to evaluate its usability in fire scenarios. The warning performance with different fire distance are displayed in Video S4. In Video S5, the fire signal is promptly sent to the mobile device. In addition, Fig. 5b also presents the thermo-voltage generated by TED-5pn and TED-10pn during multiple cycles of flame contact and long-distance exposure. The variations in thermo-voltages indicate that the designed intelligent system can achieve rapid and accurate early fire warning within a wide range of flame distances by modulating the threshold voltage or the number of p–n couples. Figure 5c depicts the promising potential of the designed TE fire warning system to achieve a next-generation remote IoT intelligent fire alarm platform, which could timely deliver warning notifications to relevant individuals or departments (such as families, communities and fire stations) to effectively prevent the spread of fires, and offering more time and opportunities to save human lives and properties. The successful application of the TE materials and TEDs for self-powered fire warning represents a significant step toward the vision of employing self-powered TE materials to achieve fast and long-life fire warning in multiple scenarios including indoor and outdoor buildings, forests, etc.

## Conclusions

In summary, a self-powered intelligent remote IoT fire warning system based on high-performance SWCNT/MXene TE composite films has been developed, displaying wide adjustable threshold voltages, ultrafast response, rapid fire detection, and long-term repeatability and durability. The optimized content ratio and effective interfacial interactions afford the flexible composite films outstanding flame retardancy and TE properties (*PF* = 239.7 ± 15.8 μW m^−1^ K^−2^), ranking the highest for SWCNT/MXene composites. Various flexible and cross-plane TED prototypes consisting of different number of p–n couples (TED-1pn, TED-5pn and TED-10pn) were assembled and comprehensively investigated for fire warning applications. The number of p–n couples, the value of the threshold voltage and the flame distance have significant effects on the fire warning performance of the TEDs. Specifically, the TED-10pn, with a threshold voltage of 1 mV, achieves an ultrafast trigger time of ~ 0.1 s, rivaling many state-of-the-art fire warning systems. Moreover, the TED-5pn demonstrates remarkable repeatability, maintaining a warning response time within 4 s after 30 cycles and within 5.5 s after 50 cycles. Notably, even after 180 days of exposure to air, the TED also retains the ability to trigger a warning response within 3 s, confirming exceptional durability, which is among the longest reported service period for TE-based fire warning materials. Finally, the designed self-powered wireless intelligent remote IoT fire warning systems, which include the TED, amplifier, ADC converter and Bluetooth module, effectively achieve fast fire detection and remotely real-time transmission of warning signals. This performance can be attributed to the high TE performance of the composite, the flexible adaptability of the number of p–n couples in TEDs to various application scenarios and the adjustable warning threshold voltage. The fire warning systems based on high-performance TE composites developed in this work show great potential in next-generation intelligent remote IoT fire warning applications.

## Supplementary Information

Below is the link to the electronic supplementary material.

Supplementary file1 (DOCX 8236 KB)

Supplementary file2 (MP4 2087 KB)

Supplementary file3 (MP4 14481 KB)

Supplementary file4 (MP4 13088 KB)

Supplementary file5 (MP4 13305 KB)

Supplementary file6 (MP4 1260 KB)
