# Supplementary material for: Ultrafast Response and Threshold Adjustable Intelligent Thermoelectric Systems for Next-Generation Self-Powered Remote IoT Fire Warning
Source: Nanomicro Lett. 2024 Jul 10;16:242. doi: 10.1007/s40820-024-01453-x (PMC11236834; doi:10.1007/s40820-024-01453-x)
Supplement: Supplementary file 1 — Supplementary file1 (DOCX 8236 KB) [file 40820_2024_1453_MOESM1_ESM.docx]

Supporting Information for

# Ultrafast Response and Threshold Adjustable Intelligent Thermoelectric Systems for Next-Generation Self-Powered Remote IoT Fire Warning

Zhaofu Ding^1^, Gang Li^1^, Yejun Wang^2^, Chunyu Du^1^, Zhenqiang Ye^1^, Lirong Liang^1,^ *, Long-Cheng Tang^2,^ * and Guangming Chen^1,^ *

^1^College of Materials Science and Engineering & College of Civil and Transportation Engineering, Shenzhen University, Shenzhen 518055, P. R. China

^2^College of Material, Chemistry and Chemical Engineering, Key Laboratory of Organosilicon Chemistry and Material Technology of MoE, Hangzhou Normal University, Hangzhou 311121, P. R. China

*Corresponding authors. E-mail: [lianglirong@szu.edu.cn](mailto:lianglirong@szu.edu.cn) (Lirong Liang); [lctang@hznu.edu.cn](mailto:lctang@hznu.edu.cn) (Long-Cheng Tang); [chengm@szu.edu.cn](mailto:chengm@szu.edu.cn) (Guangming Chen)

**Supplementary Figures and Tables**


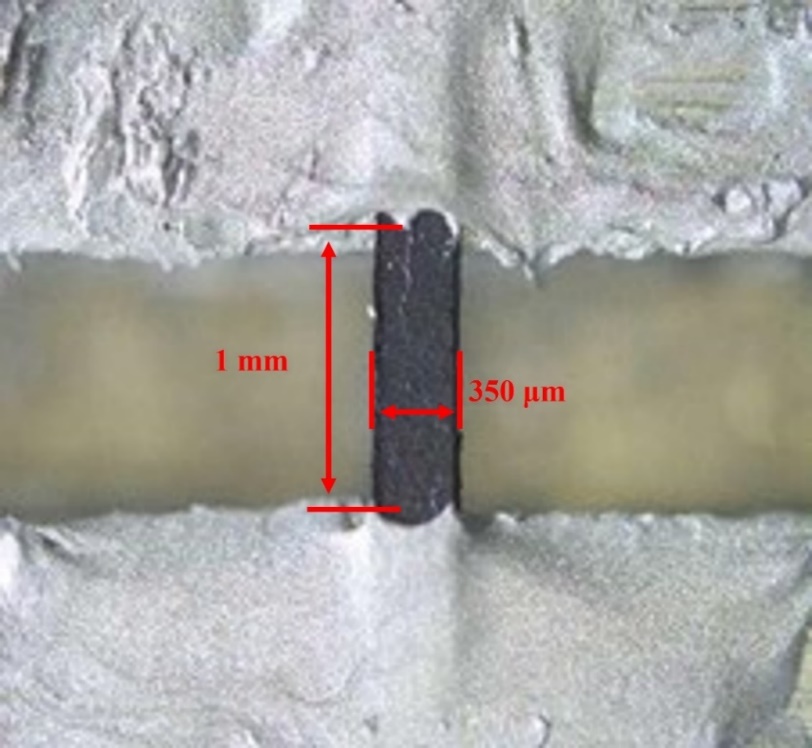


**Fig. S1** Sample size diagram for thermal diffusion coefficient test


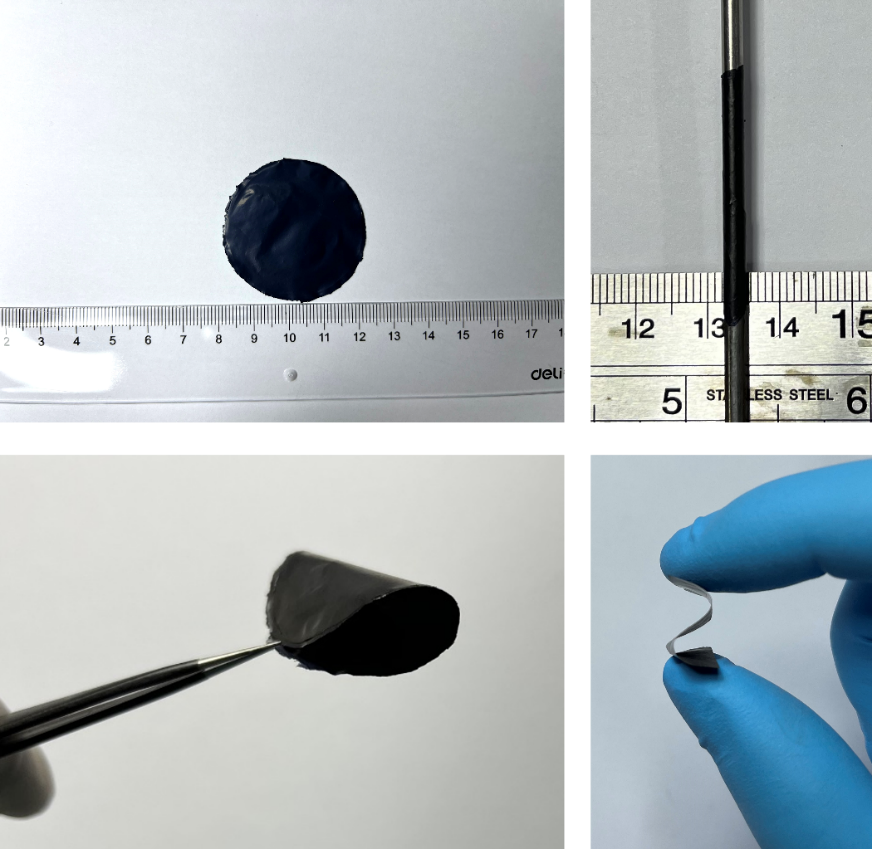


**Fig. S2** High flexibility of the SWCNT/MXene composite films with the mass ratio of 10:3


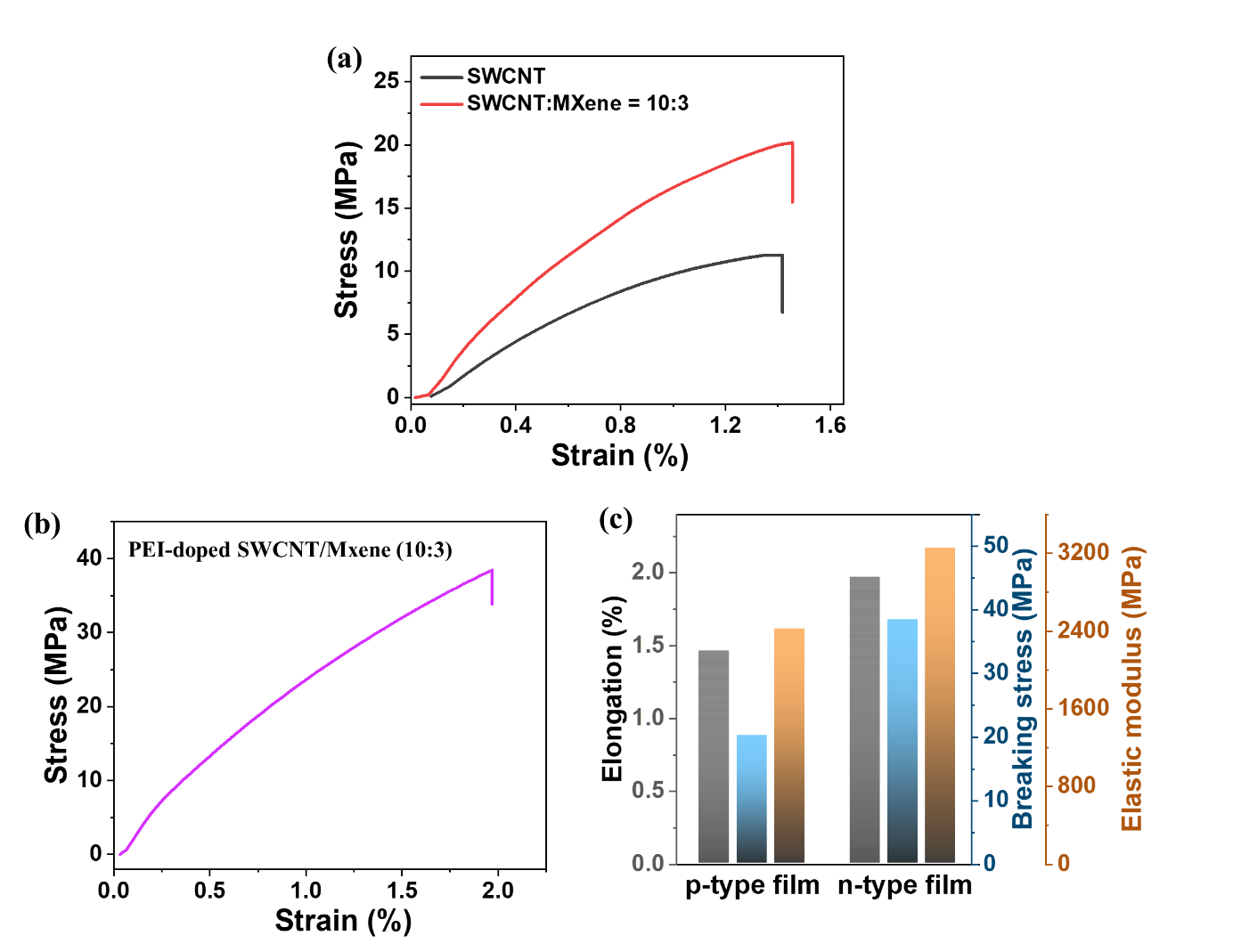


**Fig. S3** (**a**) The stress-strain curves of the pure SWCNT film, SWCNT/MXene composite films with different mass ratio. (**b**) The stress-strain curve of n-type PEI-doped SWCNT/MXene composite film with SWCNT: MXene mass ratio of 10:3


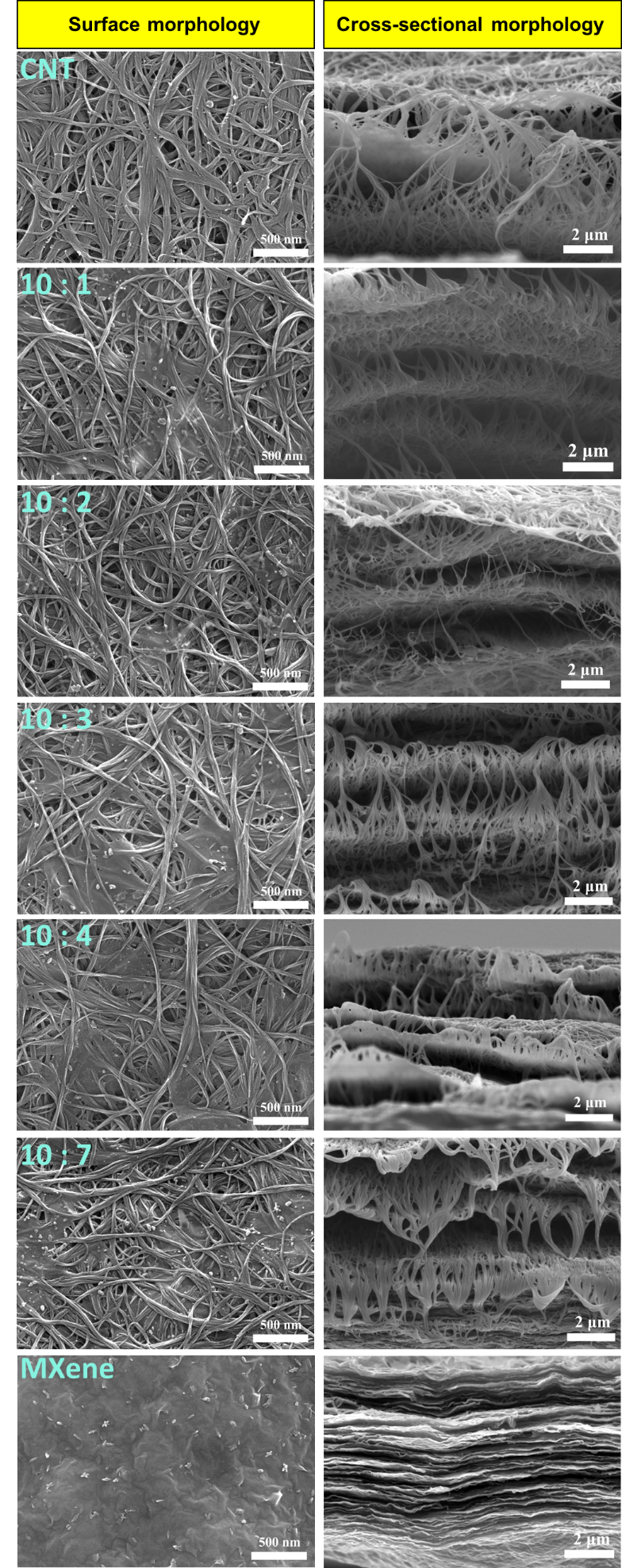


**Fig. S4** Surface and cross-sectional morphologies of the SWCNT, MXene, and their composites films with different mass ratios (SWCNT: MXene 10:1 − 10:7)


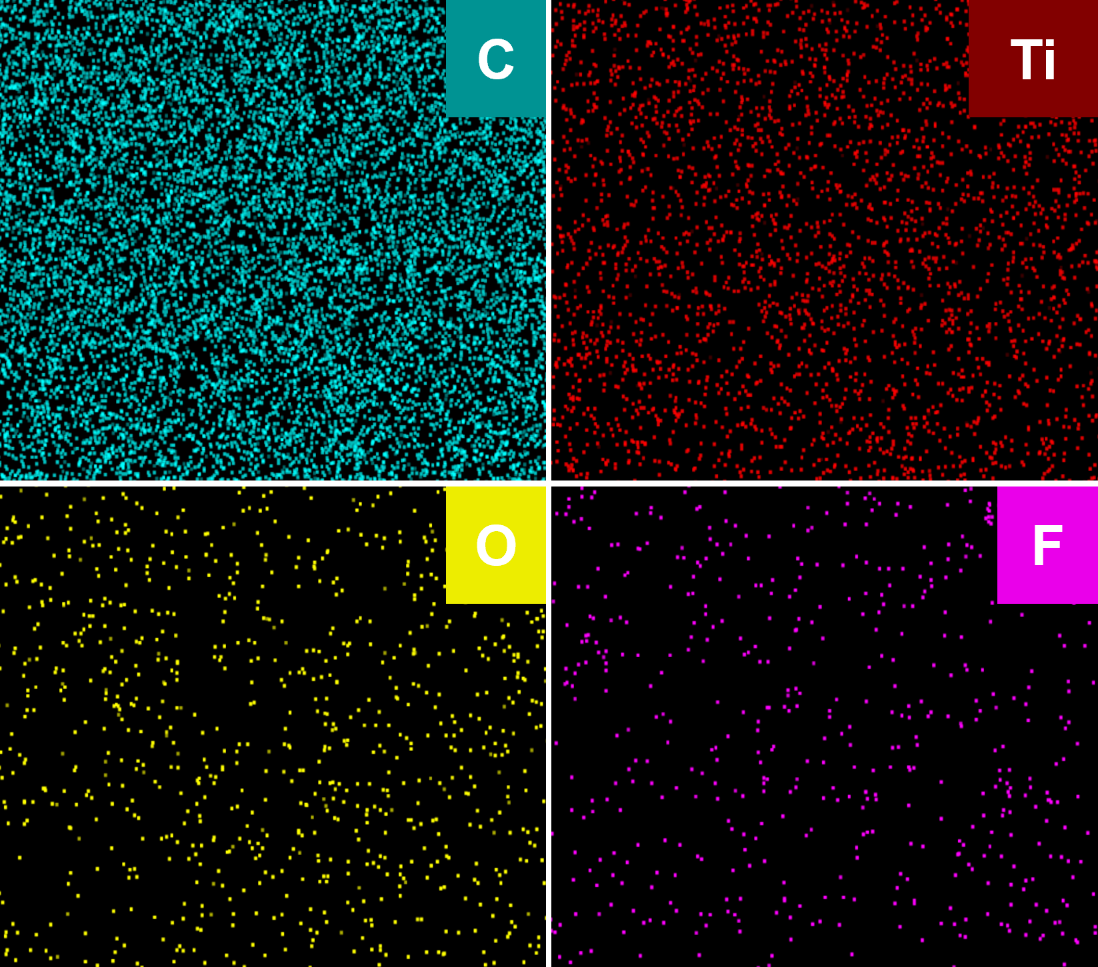


**Fig. S5** Surface SEM-EDS mappings of composite film with SWCNT to MXene mass ratio of 10:3


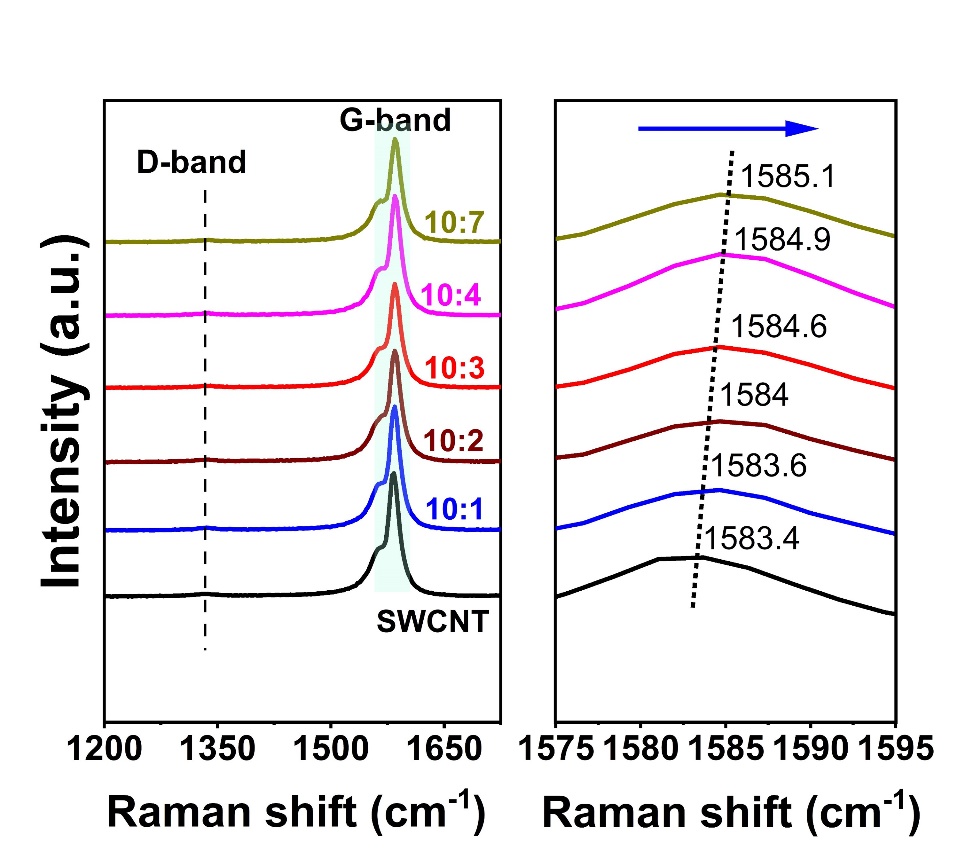


**Fig. S6** Raman spectra of pristine SWCNT/MXene composite films


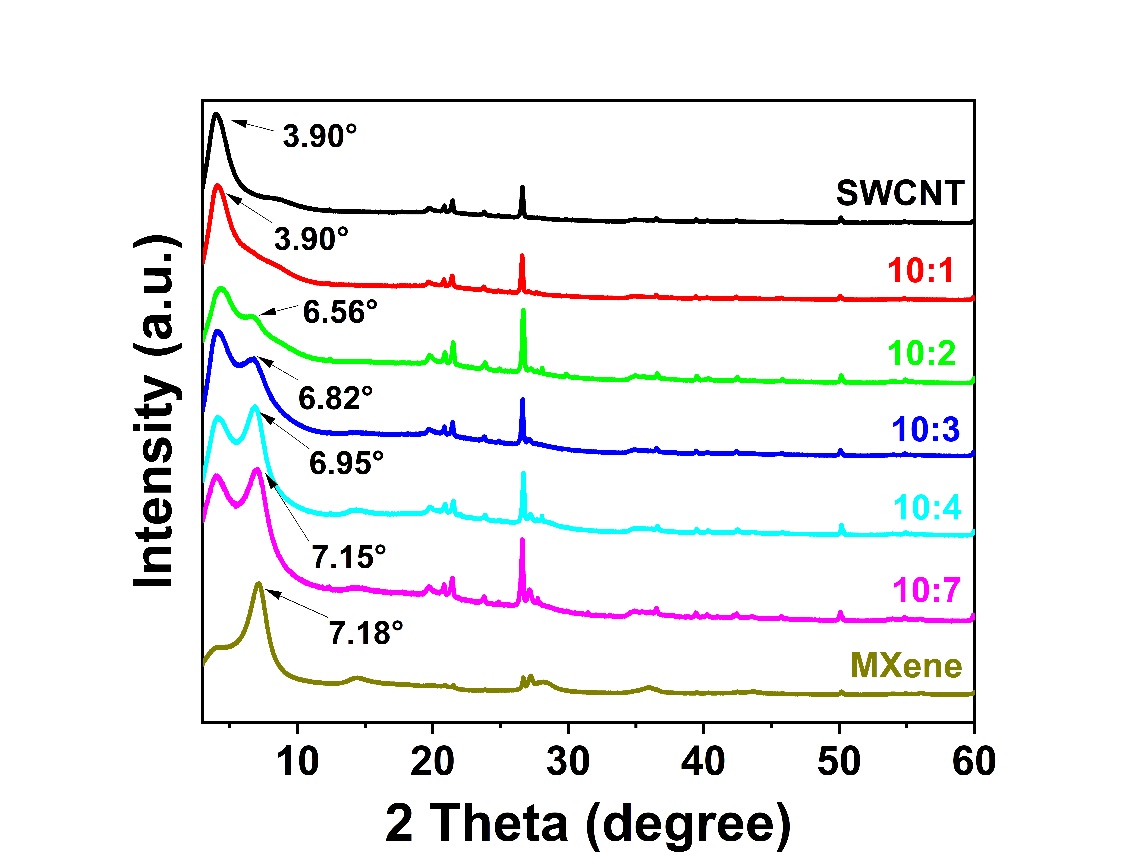


**Fig. S7** XRD patterns of SWCNT, MXene, and their composite films


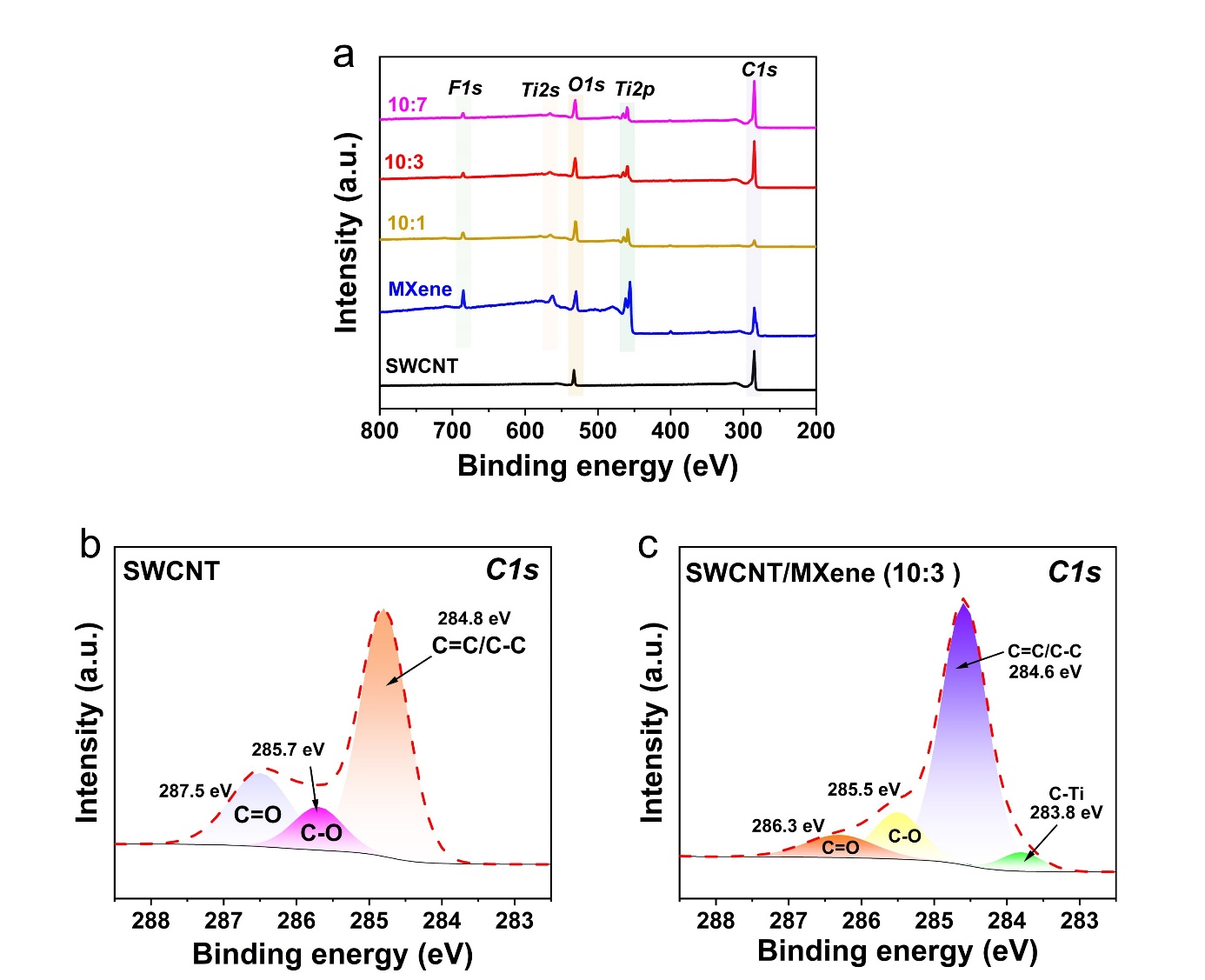


**Fig. S8** (**a**) XPS full spectra of various samples. XPS C 1s spectra of (**b**) pure SWCNT and (**c**) SWCNT/MXene composite film


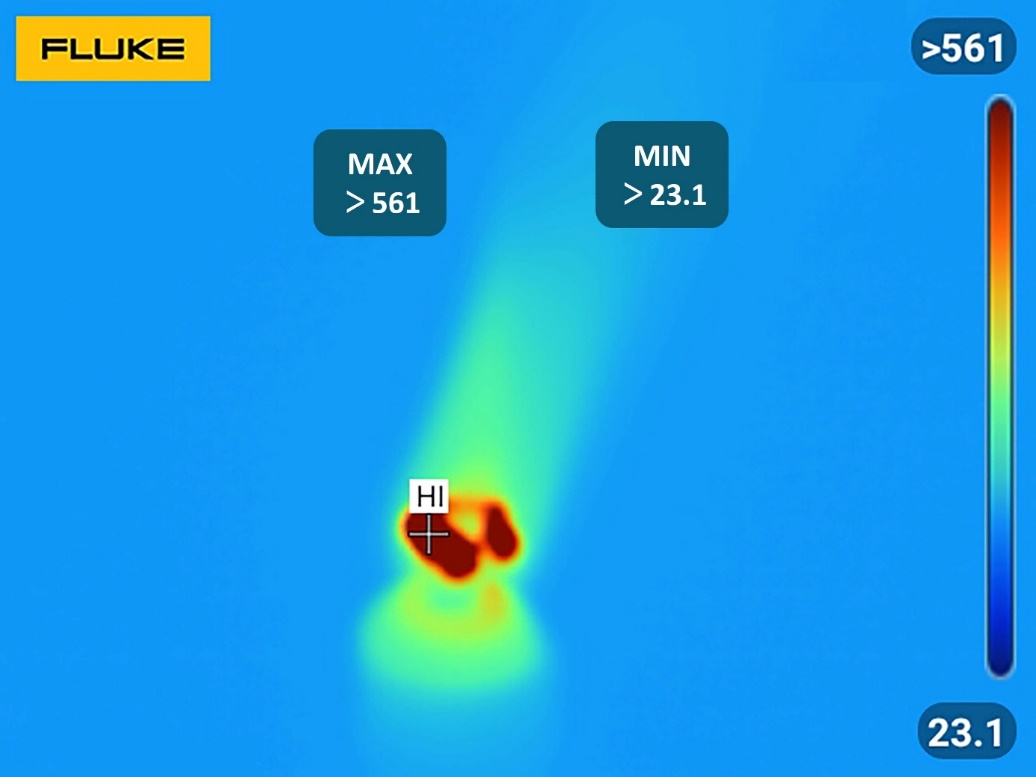


**Fig. S9** Thermal imaging of Alcohol lamp used in vertical combustion test for flame-retardant coatings samples

**
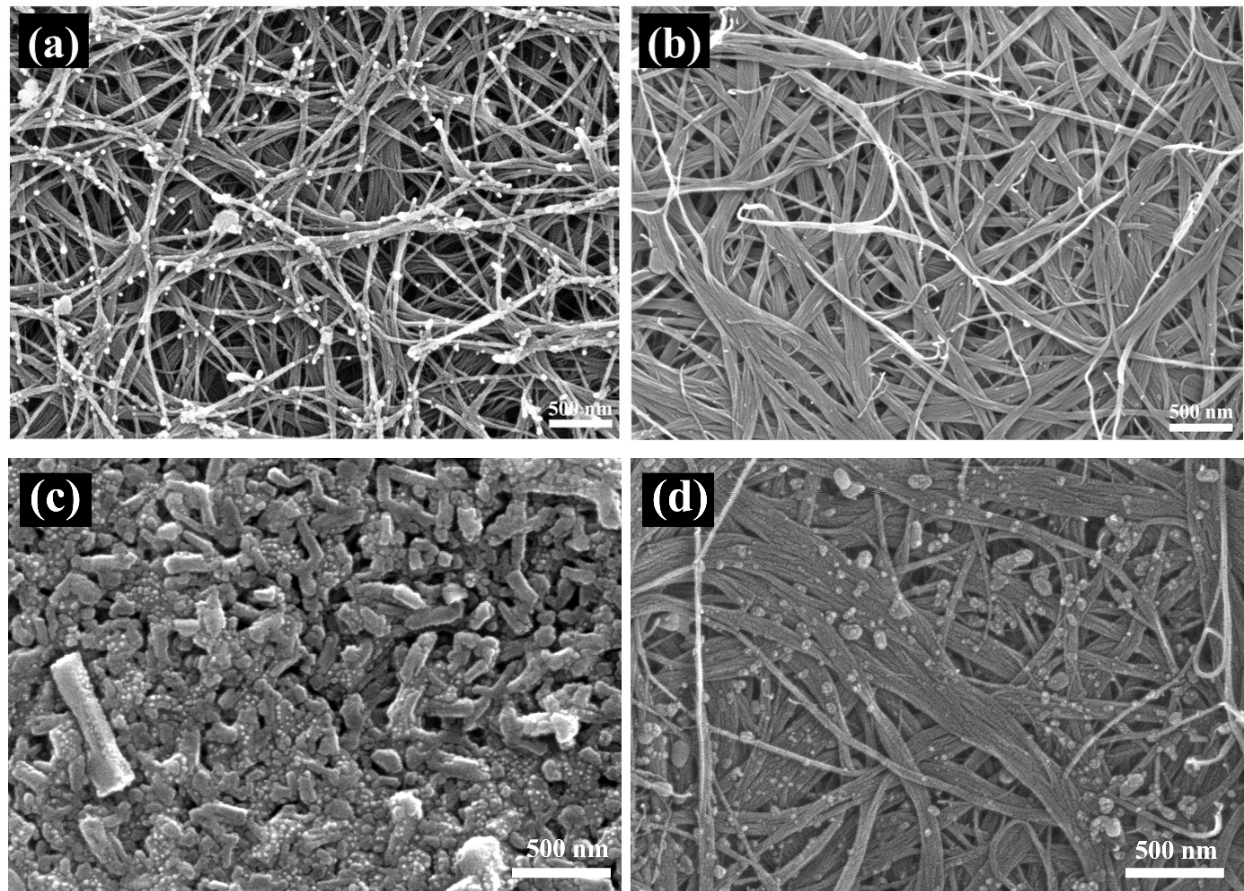
**

**Fig. S10** The surface morphology of (**a**) pure SWCNT and (**c**) SWCNT/MXene after combustion, and the internal morphology of (**b**) SWCNT and (**d**) SWCNT/MXene after the burned surface was removed with tape


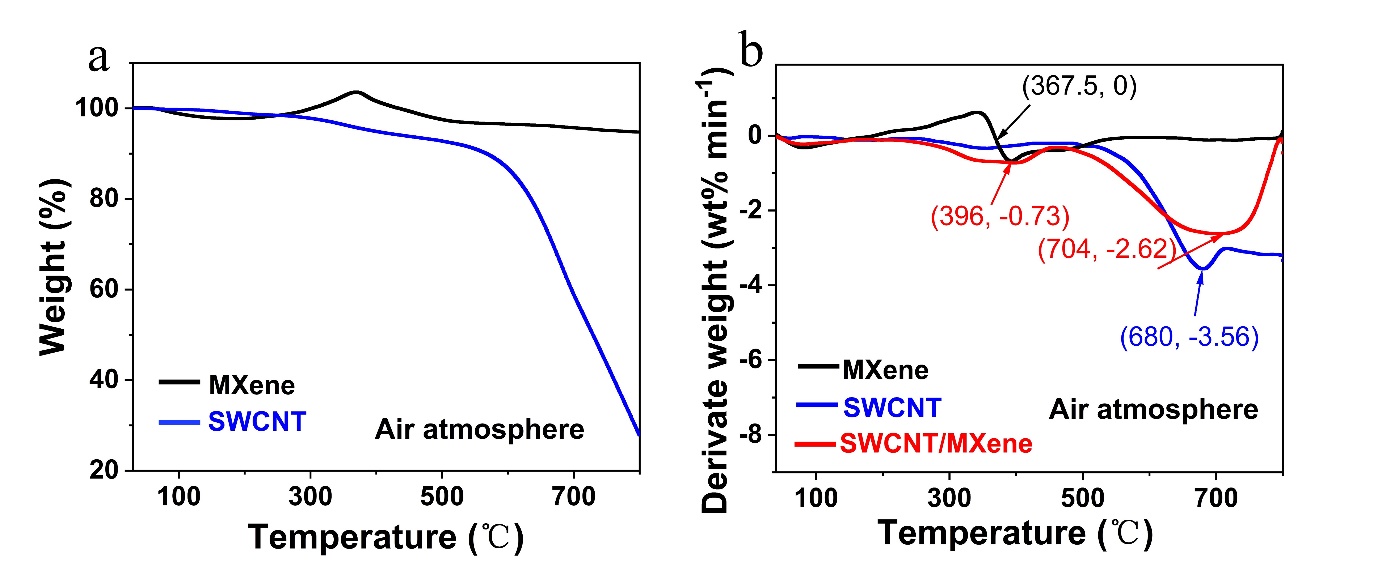
**Fig. S11** (**a**) TG curves of pure SWCNT and MXene. (**b**) DTG curves of pure SWCNT, MXene, and SWCNT/MXene 10:3 composite films under air atmosphere


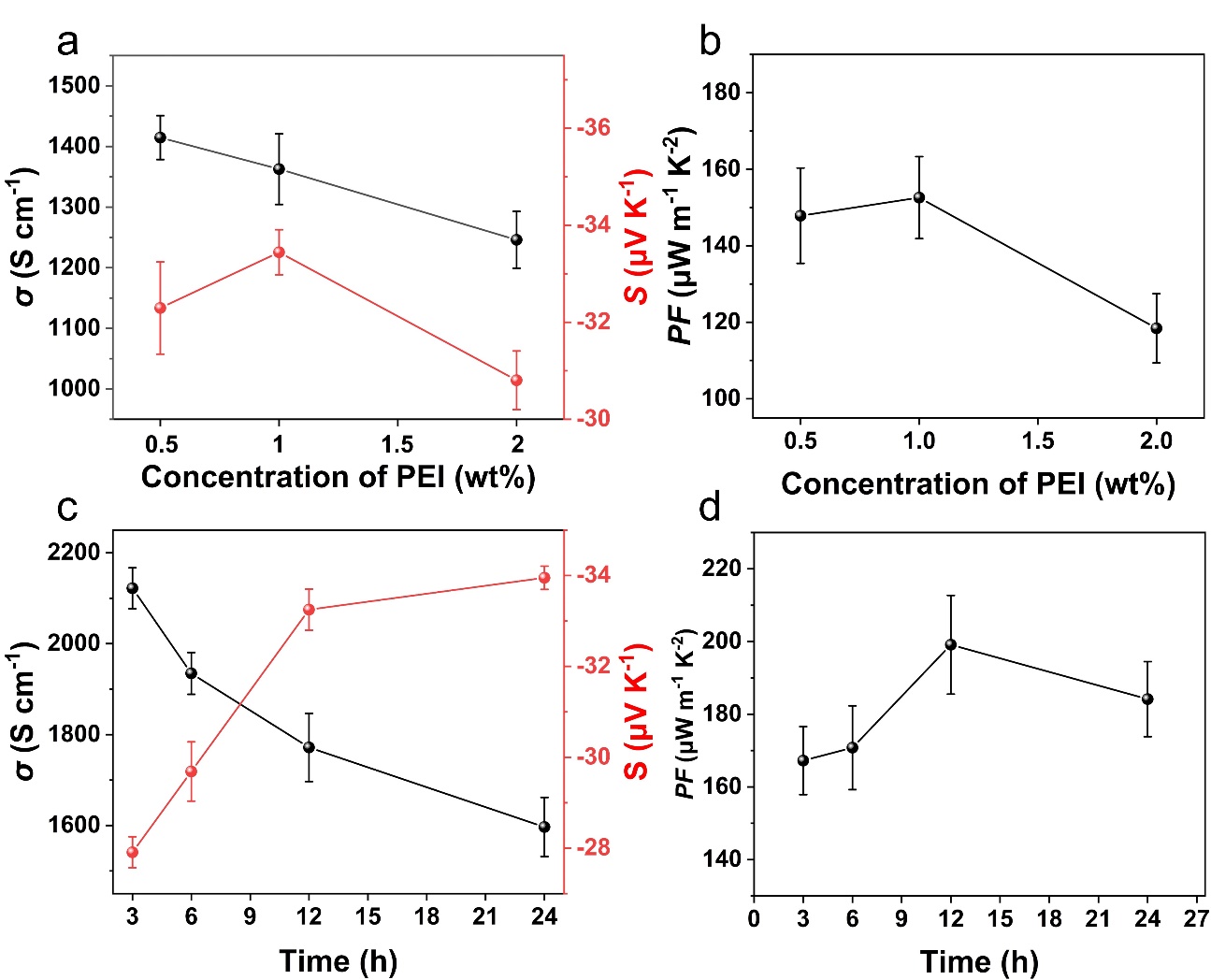


**Fig. S12** The conductivity, Seebeck coefficient and their power factor of PEI doping. (**a, b**) doping concentration, (**c, d**) soaking time

We further investigated the PEI doping concentration on the effects of TE performance. Three concentrations of PEI aqueous solutions (0.5 wt%, 1 wt%, and 2 wt%) were prepared. The doping time was constant (12 h). The corresponding electrical conductivities (*σ*), Seebeck coefficients (*S*) and calculated power factors (*PF*) were shown in **Fig.** S12a and 12b. The results show that the optimal *PF* value is obtained when PEI concentration is 1 wt%. Besides, we also have conducted the effect of doping time (3-24 h) on TE performance. The PEI concentration is constant (1 wt%). As shown in **Fig.** S12c and 12d, the best *PF* of the film was obtained when the doping time is 12 h.


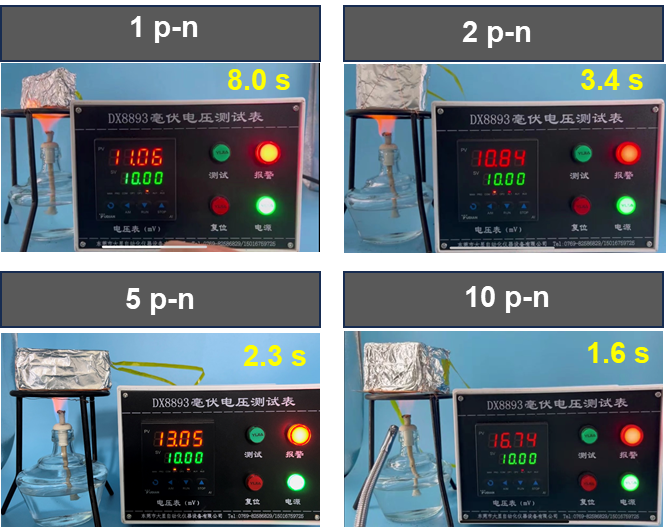


**Fig. S13** Fire warning tests for thermoelectric devices with different number of p-n couples


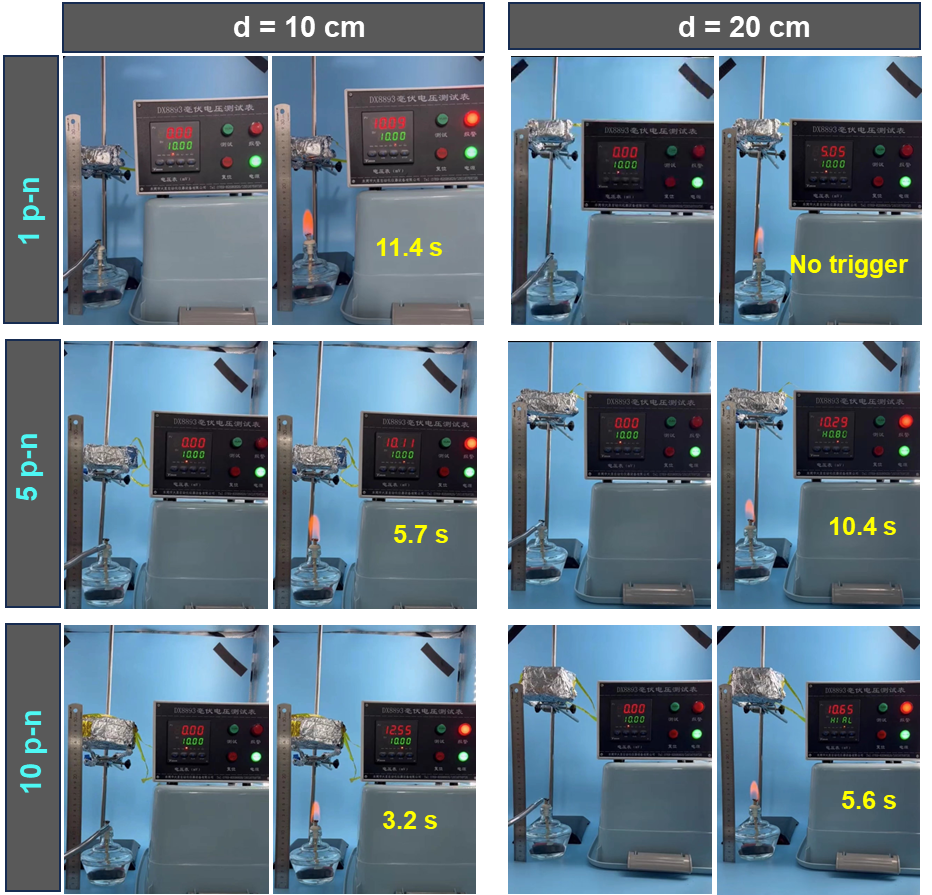


**Fig. S14** Heatwaves/temperature sensing fire warning tests for thermoelectric devices with different number of p-n couples at different flame distances


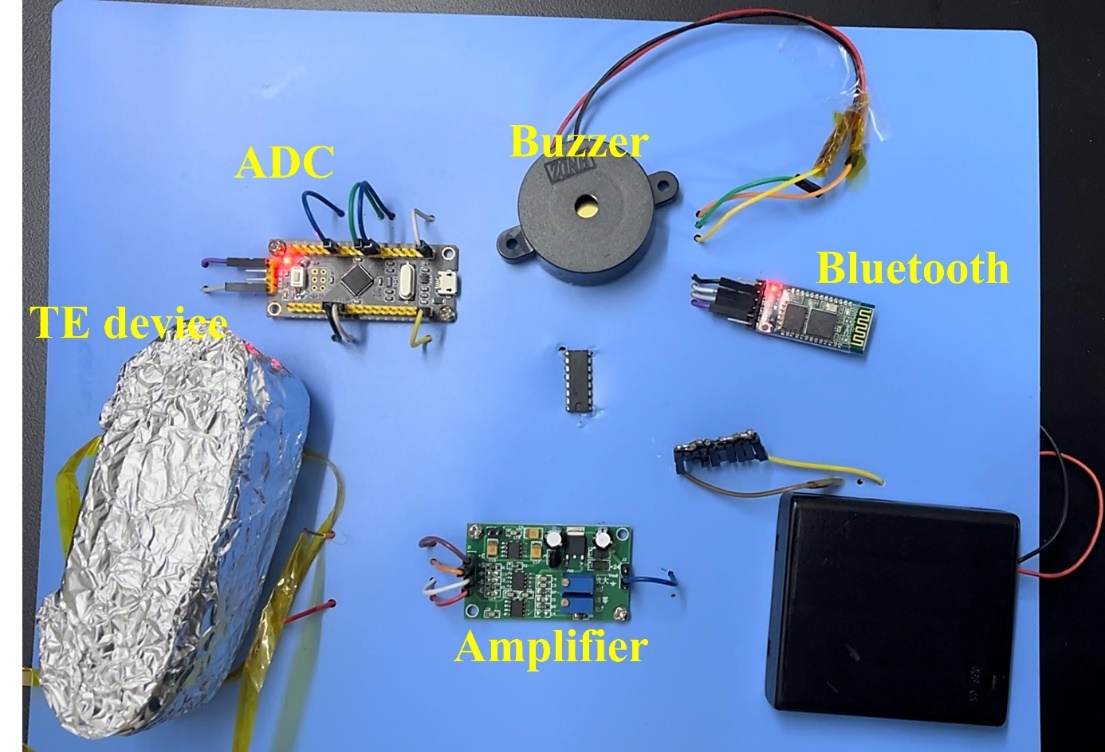


**Fig. S15** The designed self-powered smart fire warning system

**Table S1** Surface SEM-EDS elemental contents of SWCNT/MXene composite films

| **Mass ratio**  **SWCNT: MXene** | **EDS elemental mapping of SWCNT/MXene** | | | |
| --- | --- | --- | --- | --- |
|  | **C** | **Ti** | **O** | **F** |
| 10:1 | 89.01 | 2.51 | 7.13 | 1.35 |
| 10:2 | 87.37 | 3.59 | 6.91 | 2.13 |
| 10:3 | 81.37 | 5.54 | 9.59 | 3.50 |
| 10:4 | 78.05 | 8.06 | 8.77 | 5.12 |
| 10:7 | 74.68 | 13.50 | 4.61 | 7.21 |

**Table S2** Comparison of TE properties of CNT/MXene based systems

| Component | *S* (μV K^-1^) | *σ* (S cm^-1^) | *PF* (μW m^-1^ K^-2^) | | Refs. | |
| --- | --- | --- | --- | --- | --- | --- |
| SWCNT/MXene (MXene 10 wt%) | 39.64 | 1293.76 | 203.29 | [49] | |  |
| MXene/SWCNT/MXene | -32.2 | 750.9 | 77.9 | [48] | |  |
| SWCNT/MXene fiber (150-300 ℃) | 20-35 | ~80-120 | 0.179 (Max) | [36] | |  |
| SWCNT/MXene (10:3) | 43.4 | 1270.9 | 239.7 | **This work** | |  |
| PEI-dopped SWCNT/MXene (10:2) | -33.7 | 1773.5 | 202.0 |  |  |  |

**Table S3** Density (*ρ*), heat capacity (*c*_p_), thermal diffusivity (*α*), thermal conductivity (*κ*), and *ZT* value of films

| Sample | *α*  mm^2^·s^-1^ | *ρ*  g·cm^-3^ | *c*_p_  J·g^-1^·K^-1^ | *k*  W·m^-1^·K^-1^ | *ZT*  ×10^-3^ |
| --- | --- | --- | --- | --- | --- |
| SWCNT | 4.19 | 0.705 | 2.75 | 8.1233625 | 7.27 |
| p-type film | 6.08 | 0.768 | 1.65 | 7.704576 | 9.27 |
| n-type film | 9.59 | 0.868 | 0.57 | 4.749696 | 8.94 |

**Table S4** Comparison of fire warning performance of various different mechanisms

| **Mechanisms** | | **System** | **Warning time (s)** | **Threshold** | **Repeatable?** | **Long-term Stability?** | **Refs.** |
| --- | --- | --- | --- | --- | --- | --- | --- |
| Gas/smoke sensor | | Pd/SnO_2_ | ×10^1^ s | 5− 200 ppm (gas concentration) | Yes | Test for 1 month | [S1] |
| Resistant shift | | GO/Borate | 0.72 | Resistance reduced 10^4^ times | No | / | [S2] |
|  |  | Chitosan/Montmorillonite/Carbon nanotube | 0.25 | Electrical resistance 2.0 × 10^6^ Ω | No | / | [6] |
|  |  | GO/TA/P-CNFs paper | Less than 1 s | Resistance reduced 10^4^ times | No | / | [S3] |
| Chromatic change | | Phthalonitrile | Within 20 s at 275 ℃ | Color change | No | / | [8] |
|  |  | POSS-metal films | / | Color change | Yes | / | [S4] |
| Phase/shape change | | PU/MXene | Within 4 s | Shape change | No | / | [10] |
| TE | eTE | MXene/ANFs | Within 10 s | 0.1 mV | Yes | / | [31] |
|  |  | MXene/CCS | 3.8 s | 1 mV | Yes | / | [34] |
|  |  | MXene/AgNWs/ANFs | 1.6 s | 1 mV | Yes | / | [58] |
|  |  | PI/MXene | 4.67 s | 1 mV | Yes | / | [33] |
|  |  | PI/MXene/Ag2Se | 4.03 s | 1 mV | Yes | / | [32] |
|  |  | MXene/TA/CaCl2 | Within 3 s | 4 mV | Yes | / | [11] |
|  |  | PPy/MMT/APP | 1 s | 5 mV | Yes |  | [17] |
|  |  | CNF/MXene | 1 s | 1 mV | Yes | / | [S5] |
|  |  | MXene/UPC/MMT | 3.1 s | 1 mV | Yes | / | [35] |
|  |  | SWCNT/MXene | 1.43 s | 1 mV | Yes | / | [36] |
|  |  | SWCNT/PEDOT:PSS | 2.1 s | 1 mV | Yes |  | [13] |
|  |  | SWCNT/MXene | ~0.1 s | 1 mV | Excellent | Test for 6 months | **This work** |
|  |  |  | 0.7 s | 5 mV |  |  |  |
|  |  |  | 1.6 s | 10 mV |  |  |  |
|  | iTE | Phosphorous-containing ionic liquid | 1.1 s | 50 mV | Yes | / | [29] |
|  |  | Ionic hydrogel | ~4 s | 100 mV | Yes | / | [30] |
|  |  | Temperature-arousing battery | 79 s (practical tests) | / | / | / | [S6] |

**Video S1** Flame retardancy of the SWCNT/MXene (S/M) composite film and coating

**Video S2** Representative fire warning tests with the 1^th^, 30^th^ and 50^th^ repeated warning performance

**Video S3** Fire warning test with TED-10pn in fire directly, 10 cm and 20 cm fire distance, respectively

**Video S4** Building fire warning tests at flame distances of 0, 10, and 20cm, respectively

**Video S5** Fire hazard signals were sent through mobile devices

**Supplementary References**

1. L. Xiao, S. Xu, G. Yu, S. Liu, Efficient hierarchical mixed Pd/SnO_2_ porous architecture deposited microheater for low power ethanol gas sensor. Sens. Actuators B Chem. **255**, 2002-2010 (2018). [https://doi.org/10.1016/j.snb.2017.08.216](https://doi-org.ezproxy.lib.szu.edu.cn/10.1016/j.snb.2017.08.216)
2. C. F. Cao, B. Yu, J. Huang, X. L. Feng, L. Y. Lv et al., Biomimetic, mechanically strong supramolecular nano system enabling solvent resistance, reliable fire protection and ultralong Fire Warning. ACS Nano **16**, 20865-20876 (2022). <https://doi.org/10.1021/acsnano.2c08368>
3. C.-F. Cao, B. Yu, B.-F. Guo, W.-J. Hu, F.-N. Sun et al., Bio-inspired, sustainable and mechanically robust graphene oxide-based hybrid networks for efficient fire protection and warning Chem. Eng. J. **439**, 134516 (2022). <https://doi.org/10.1016/j.cej.2022.134516>
4. . Xu, L. Huang, J. Long, R. Zhang, Z. Zhong et al., Reversible thermochromic POSS-Metal Films for early warning. Compos. Sci. Technol. **217**, 109083 (2022). https://doi. org/10.1016/j.compscitech.2021.109083
5. H. Wang, Y. Jiang, Z. Ma, Y. Shi, Y. Zhu et al., Hyperelastic, robust, fire-safe multifunctional MXene aerogels with unprecedented electromagnetic interference shielding efficiency. Adv. Funct. Mater. **33**, 2306884 (2023). <https://doi.org/10.1002/adfm.202306884>
6. X. Li, S. Lyu, J. Jia, N. Gao, X. Wu et al., A bio-Inspired temperature-arousing battery with giant power for fire alarming. Adv. Funct. Mater. **33**, 2300403 (2023). <https://doi.org/10.1002/adfm.202300403>
